# GABAergic interneurons form transient layer-specific circuits in early postnatal neocortex

**DOI:** 10.1038/ncomms10584

**Published:** 2016-02-04

**Authors:** Paul G. Anastasiades, Andre Marques-Smith, Daniel Lyngholm, Tom Lickiss, Sayda Raffiq, Dennis Kätzel, Gero Miesenböck, Simon J. B. Butt

**Affiliations:** 1 Department of Physiology, Anatomy and Genetics, University of Oxford, South Parks Road, Oxford OX1 3QX, UK; 2 Centre for Neuroscience, Imperial College Faculty of Medicine, Hammersmith Hospital, Imperial College London, London W12 0NN, UK; 3 Department of Clinical and Experimental Epilepsy, Institute of Neurology, University College London, Queen Square, London WC1N 3BG, UK; 4 Centre for Neural Circuits and Behaviour, University of Oxford, Oxford OX1 3SR, UK

## Abstract

GABAergic interneurons play key roles in cortical circuits, yet little is known about their early connectivity. Here we use glutamate uncaging and a novel optogenetic strategy to track changes in the afferent and efferent synaptic connections of developing neocortical interneuron subtypes. We find that *Nkx2-1*-derived interneurons possess functional synaptic connections before emerging pyramidal cell networks. Subsequent interneuron circuit maturation is both subtype and layer dependent. Glutamatergic input onto fast spiking (FS), but not somatostatin-positive, non-FS interneurons increases over development. Interneurons of both subtype located in layers (L) 4 and 5b engage in transient circuits that disappear after the somatosensory critical period. These include a pathway mediated by L5b somatostatin-positive interneurons that specifically targets L4 during the first postnatal week. The innervation patterns of immature cortical interneuron circuits are thus neither static nor progressively strengthened but follow a layer-specific choreography of transient connections that differ from those of the adult brain.

## Abstract (web summary)

GABAergic interneurons are key components of cortical circuits, yet their early connectivity is unknown. Here the authors show that during early postnatal development, *Nkx2-1*-derived interneurons engage in layer-specific and dynamic circuits, which are distinct from those in the mature neocortex.

## Graphical abstract

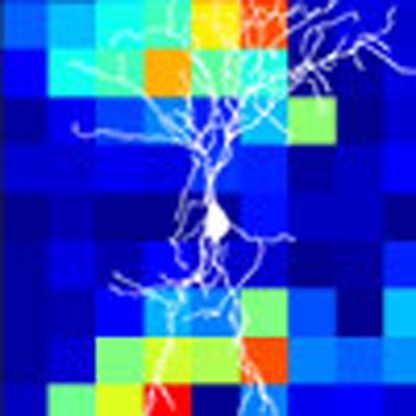

GABAergic interneurons are important for the development^1^,^2^ and mature function of the cerebral cortex. Cognitive processing depends on the function of a heterogeneous array of GABAergic interneurons^3^, which provide subtype-specific innervation of other interneurons^4^, as well as different somatodendritic compartments of excitatory pyramidal projection neurons^5^. In contrast to our understanding of interneuron diversity in the adult cortex, the role of different subtypes during development is less well established, even though evidence from genetic fate-mapping experiments suggest that the basic logic of the inhibitory system is established early^6^,^7^,^8^.

Detailed knowledge of early interneuron diversity and synaptic connectivity is of particular importance given growing evidence that molecular deficits which interfere with the development and synaptic integration of GABAergic interneurons might play a role in a number of neurodevelopmental disorders^9^. It is likely that such deficits have knock-on effects via the pivotal role of inhibition in developing networks^1^,^10^. In the early postnatal brain, it is evident that interneurons are important contributors to at least two key phases of circuit formation. First, GABAergic activity, in the form of giant depolarizing potentials (GDPs), represents a key mechanism for synchronizing neurons during the early period of synaptogenesis^11^. Second, GABAergic signalling can influence circuit refinement in response to sensory experience^2^,^12^,^13^. The evolving roles of GABAergic transmission appear to correlate with the emergence of distinct interneuron subtypes. Hub cells, which are critical for the generation of GDPs, consist of early born, somatostatin (SST)-positive, dendrite-targeting interneurons^14^, while sensory refinement and critical period plasticity are thought to coincide with the relatively late maturation of parvalbumin (PV)-expressing basket cells. Yet intriguingly the interneuron subtypes that are linked to these critical early network events share a common spatial origin in the *Nkx2-1*-expressing ventricular zone (VZ) of the embryonic ventral telencephalon^15^. While a number of studies have demonstrated that embryonic origin is predictive of mature interneuron subtype, remarkably little is known about the timeline over which interneuron subtypes form afferent and efferent synaptic connections beyond their immediate layer^16^.

To understand better the contribution of interneuron diversity to emergent cortical function, we have tracked the local network integration of *Nkx2-1*-derived GABAergic interneurons over a developmental time frame that encompasses both neocortical GDP generation^17^ and the critical period in somatosensory barrel cortex^2^. To follow the integration of *Nkx2-1*-derived interneurons, we have employed laser scanning photostimulation (LSPS)^18^,^19^, an approach that has previously been used to great effect to reveal the laminar and columnar organization of connections from pyramidal cells (PYRs) onto interneurons in the adult neocortex^20^,^21^,^22^,^23^. In a second series of experiments, we have identified the postsynaptic PYR targets of the same cohort of interneuron using a novel optogenetic circuit-mapping approach^24^. The combination of these two methods revealed the existence of prominent, but in some cases transient, translaminar synaptic connections between *Nkx2-1* interneurons and PYRs during key periods of cortical network formation and plasticity.

## Results

### Identification of *Nkx2-1* interneurons during early development

**Figure 1:**
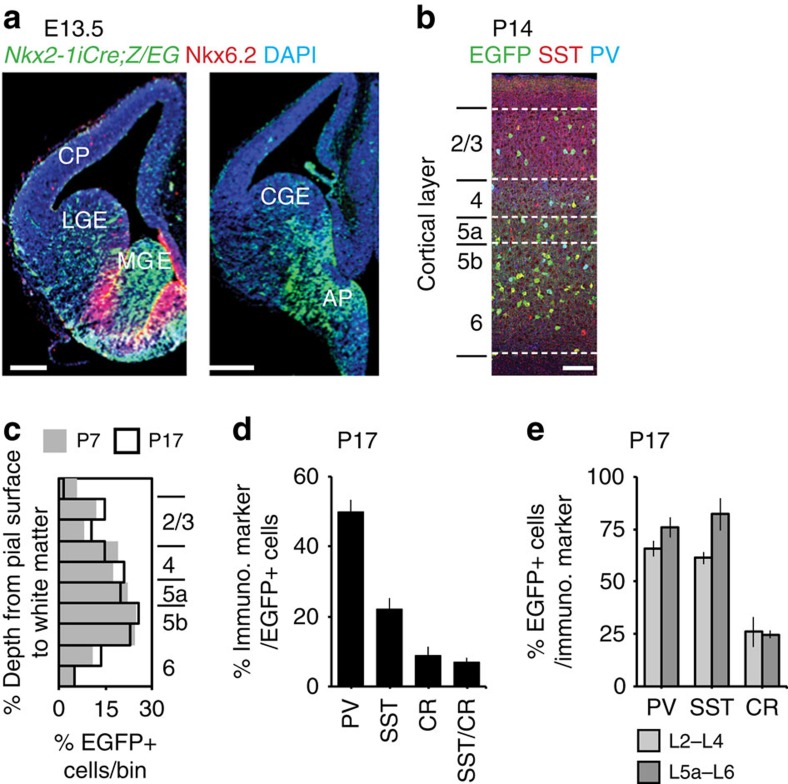
Origin and identity of early postnatal *Nkx2-1* neocortical interneurons. (**a**) EGFP expression in rostral (left) and more caudal (right) aspect of the embryonic (E)13.5 telencephalon. There was an absence of EGFP in Nkx6.2-expressing progenitors (red) bordering the sulcal region between the MGE and lateral ganglionic eminence. AP, anterior entopeduncular area; CP, cortical plate; MZ, marginal zone; WM, white matter tract. Scale bar, 100 μm. (**b**) Distribution of EGFP+ cells across the depth of the whisker barrel cortex at P14. (**c**) Comparison of the distribution of *Nkx2-1*-derived interneurons at early (P7) and more mature (P17) ages (data sampled from *n*≥4 brains). (**d**) Percentage of EGFP+ cells expressing parvalbumin (PV; sampled from *n*=5 brains), somatostatin (SST; *n*=5), calretinin (CR; *n*=7) or both SST and CR (SST/CR; *n*=5) measured across the depth of an arbitrary 250 μm column in S1BF at P17. (**e**) Percentage of cells expressing any given marker that also expressed EGFP at P17, split according to superficial (layers (L)2–4) and deep (L5a–6) layers. All data in **d**,**e** are mean±s.e.m.

We employed a conditional fate-mapping approach to identify and track the development of a cohort of GABAergic interneurons, termed *Nkx2-1* interneurons, in the neocortex of *Nkx2-1 iCre;Z/EG* mice. Consistent with previous findings^25^, this approach resulted in enhanced green fluorescent protein (EGFP) expression in the neuroepithelium of the medial ganglionic eminence (MGE) and preoptic area (Fig. 1a) but exclude progenitors of the caudal ganglionic eminence (CGE), and cells bordering the sulcus between the MGE and lateral ganglionic eminence, where *Nkx2-1* and *Nkx6.2* are co-expressed (Fig. 1a). The distribution of EGFP+ cells in the mouse somatosensory whisker barrel cortex (S1BF) exhibited a location bias towards granular and infragranular layers (L5a–6; Fig. 1b) throughout early postnatal development (postnatal day (P)3–17), and displayed little change ≥P7 (Fig. 1c).

**Figure 2:**
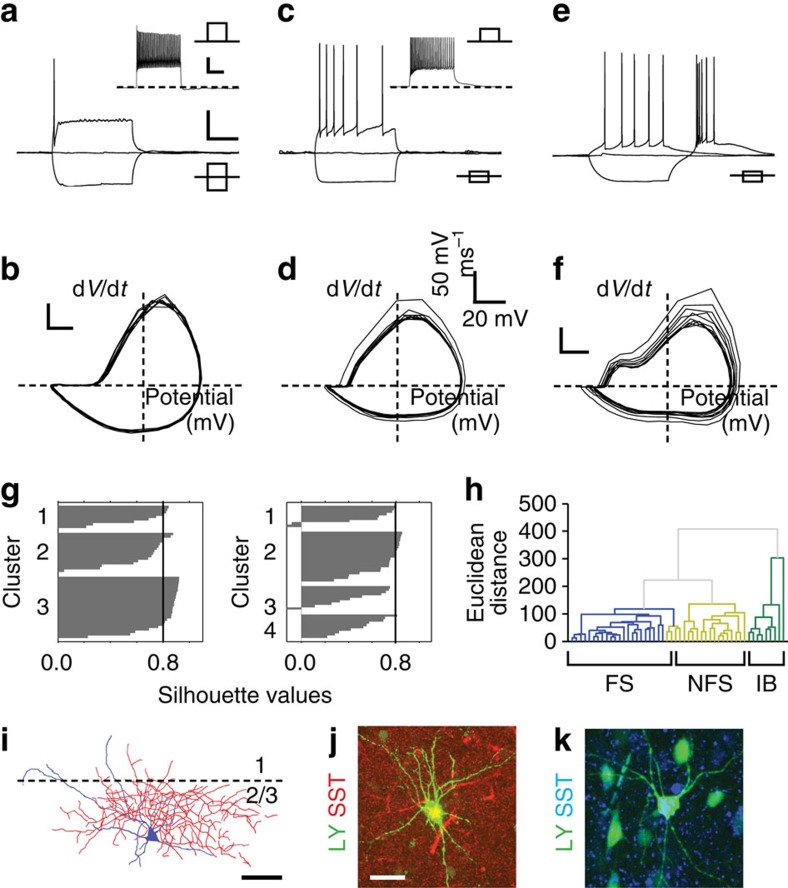
Electrophysiological profile of mature *Nkx2-1* neocortical interneuron subtypes. Intrinsic electrophysiology profiles of the three observed *Nkx2-1* interneuron subtypes: (**a**,**b**) fast spiking (FS), (**c**,**d**) non-fast spiking (NFS) and (**e**,**f**) rebound intrinsic bursting (rIB) interneuron. Scale bar (shown in **a**): 200 ms, 25 mV (**a**,**c**,**e**) Threshold spike, resting membrane potential and response to hyperpolarising current injection for the various subtypes; note the burst of two or more action potentials observed in response to release of hyperpolarising current injection in the rIB interneuron (**e**). Inset: corresponding near maximal firing frequency response for the (**a**) FS, and (**c**) NFS cells; rIB interneurons showed adaptation in spike frequency similar to the (**c**) NFS subtype. (**b**,**d**,**f**) Phase plot (d*V*/d*t* versus voltage) for the cells shown in **a**, **c** and **e**, observed in response to suprathreshold current injection sufficient to elicit 10 spikes (20 Hz). (**g**) Silhouette plot derived from *k*-means (Matlab R2014a) cluster analysis with *k* (the number of clusters) set at 3 (left) and 4 (right plot); larger silhouette values closer to 1 are indicative of a compact cluster distinct from other clusters. (**h**) Dendrogram of hierarchical unsupervised clustering based on nine electrophysiological variables measured in 50 *Nkx2-1* interneurons. The *x* axis represents individual cells and the *y* axis Euclidean distance. FS cells are shown in blue, NFS and rIB in light and dark green, respectively. (**i**) Dense multipolar arbour of the P16 FS cell shown in **a**,**b**; scale bar, 28 μm. (**j**,**k**) Somatostatin expression in recovered lucifer yellow (LY)-filled NFS (**j**) and rIB (**k**) interneuron; LY-filled neurons (arrows) were distinguished from other *Nkx2-1* interneuron GFP+ profiles by their extensive dendritic and axon fills. Scale bar, 15 μm.

To examine the diversity of interneuron subtypes represented by the *Nkx2-1* cohort, we stained the P17 EGFP+ population for a number of interneuron markers (PV; SST; calretinin; Fig. 1d,e). The percentages of EGFP+ cells expressing these markers were similar to a previous fate-mapping study^25^ (Fig. 1d). However, our counts of cells expressing any given marker that were also EGFP+ (Fig. 1e), indicated that we were not capturing the entire population often attributed to the MGE^15^. Therefore, to further characterize the diversity of interneurons in our genetic sample, we recorded the intrinsic electrophysiological properties and reconstructed the morphologies of EGFP+ cells in acute *in vitro* cortical slices (Fig. 2). Mature (P13–21) EGFP+ cells comprised a number of distinct electrophysiological subtypes: (i) fast spiking (FS; *n*=40) interneurons that showed no adaptation in spike frequency (Fig. 2a,b); (ii) non-FS (NFS), predominantly type-1 (ref. 15), adapting interneurons (*n*=23; Fig. 2c,d); and (iii) rebound intrinsic bursting (rIB) cells (*n*=14; Fig. 2e,f). K-means (Fig. 2g) and hierarchical (Fig. 2h) cluster analysis of intrinsic electrophysiological parameters (Methods) identified the presence of three distinct electrophysiological populations. Recovered FS cells were SST-negative, multipolar basket cells with predominantly locally projecting, intralaminar axonal arbours (Fig. 2i). The other two populations consisted of SST-positive, bitufted (Fig. 2j,k) Martinotti cells. FS and NFS interneurons were distributed across all layers of the neocortex, while rIBs were confined to infragranular layers, primarily L5b/6 (13/14 cells). Together, these data suggest that our genetic strategy (*Nkx2-1iCre;Z/EG*) captures a cohort of GABAergic interneuron that is not as diverse as that previously reported for the anatomically defined MGE^26^ and as such more amenable for a longitudinal study during early postnatal development.

**Figure 3:**
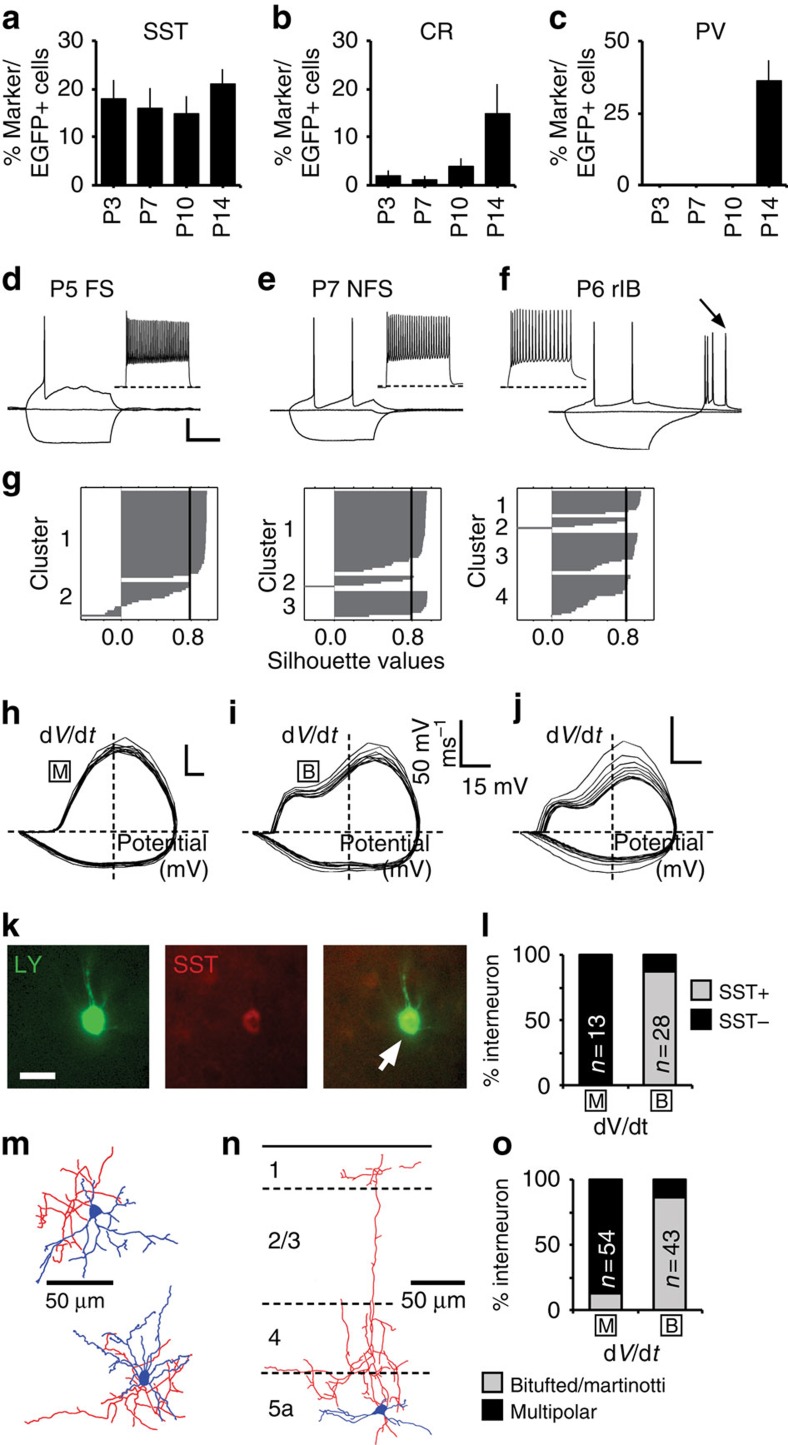
Identification of Nkx2-1 interneuron subtypes through early postnatal development. (**a**) Percentage of *Nkx2-1Cre;Z/EG* EGFP+ cells expressing somatostatin (SST) across the depth of a 250-μm width column of S1BF neocortex at four time points through early development; corresponding data for (**b**) calretinin (CR) and (**c**) parvalbumin (PV; data obtained from n≥4 brains). Responses to threshold and hyperpolarizing current injection of **d** P5 fast spiking (FS) interneuron; (**e**) P7 non-fast spiking (NFS) interneuron; (**f**) P6 rebound intrinsic spiking interneuron (rIB), arrow, burst of action potentials observed following release from hyperpolarizing current injection; scale bar, 20 mV, 200 ms. Inset: near maximal firing frequency recordings for the corresponding cells. (**g**) Silhouette plot derived from *k*-means cluster analysis with *k* (the number of clusters) set at 2 (left), 3 (middle) and 4 (right plot). (**h**–**j**) Phase plot (d*V*/d*t* versus voltage) for the same cells as **d**–**f** observed in response to suprathreshold current injection sufficient to elicit 10 spikes (20 Hz). [B], biphasic rising phase; [M], monophasic rising phase. (**k**) SST expression (arrow) in the recovered Lucifer yellow (LY)-filled soma of the neuron shown in **e** (scale bar, 12 μm). (**l**) Relationship between phase plot profile (*x* axis; M, monophasic, B, biphasic) and expression of SST in recorded immature interneurons. (**m**,**n**) Reconstructed morphologies of early *Nkx2-1* interneurons with characteristic multipolar, local arbor, SST-negative, immature FS interneurons. (**n**) Distinctive ascending axon of a SST-expressing immature NFS interneuron. Approximate layer boundaries indicated by the dashed lines. (**o**) Histogram showing the percentage of cells assigned to a given phase plot profile (*x* axis) that were classified as either bitufted dendritic morphology (grey bar), often with an ascending axons (Martinotti), or multipolar dendrites (black; typically ≥4 primary dendrites).

To enable discrimination of interneuron subtypes at earlier postnatal ages, we extended our immunohistochemical, electrophysiological and morphological analyses to all time points from P1 onwards (Fig. 3). The most consistent immunohistochemical marker and therefore reliable identifier of subtype was SST, which labelled a fifth of EGFP+ cells throughout early development (Fig. 3a), with a small number of cells in L2/3 found to co-express calretinin at later ages (Fig. 3b)^27^. The late developmental expression of the other principal marker of *Nkx2-1* interneurons, PV (Fig. 3c), made it unsuitable for use before P14. To examine the intrinsic electrophysiological and morphological characteristics of immature *Nkx2-1* interneurons, we recorded intrinsic electrophysiological profiles from a further 176 EGFP+ cells (<P13; Fig. 3d–f). K-means (Fig. 3g) cluster analysis was inconclusive, with the majority of cells falling into a single cluster. Our inability to discriminate subtypes based on intrinsic electrophysiological properties likely reflected the immature profile of interneurons at these ages and the progressive maturation of a number of key diagnostic parameters over the time period studied (Supplementary Fig. 1; Supplementary Tables 1–3)^28^. Therefore, for any given cell, the most effective classification strategy was to combine action potential phase plots (Fig. 3h–j)^29^ with the expression of SST (Fig. 3k,l) and morphological criteria (Fig. 3m–o). This approach enabled us to identify immature FS interneurons at the same frequency (*n*=72/132) as observed ≥P13. Although rebound bursting—the key diagnostic criterion for rIB cells—was recorded in a few fate-mapped EGFP+ cells at immature ages (*n*=8; Fig. 3f), rIB cells were not observed as commonly as at mature ages. The late maturation of this single defining intrinsic electrophysiological property meant that some putative early rIB SST+ cells—which are in every other aspect indistinguishable from corresponding immature NFS, SST+ interneurons (Supplementary Tables 1 and 2)—were likely included in the NFS sample (*n*=52). This limitation notwithstanding, we were able to distinguish reliably between two *Nkx2-1* populations: SST-negative, FS basket cells and SST+, non-FS interneurons. This allowed us to investigate the synaptic integration of these interneuron types into cortical circuits throughout early postnatal development.

### *Nkx2-1* cells acquire glutamatergic afferent input early

**Figure 4:**
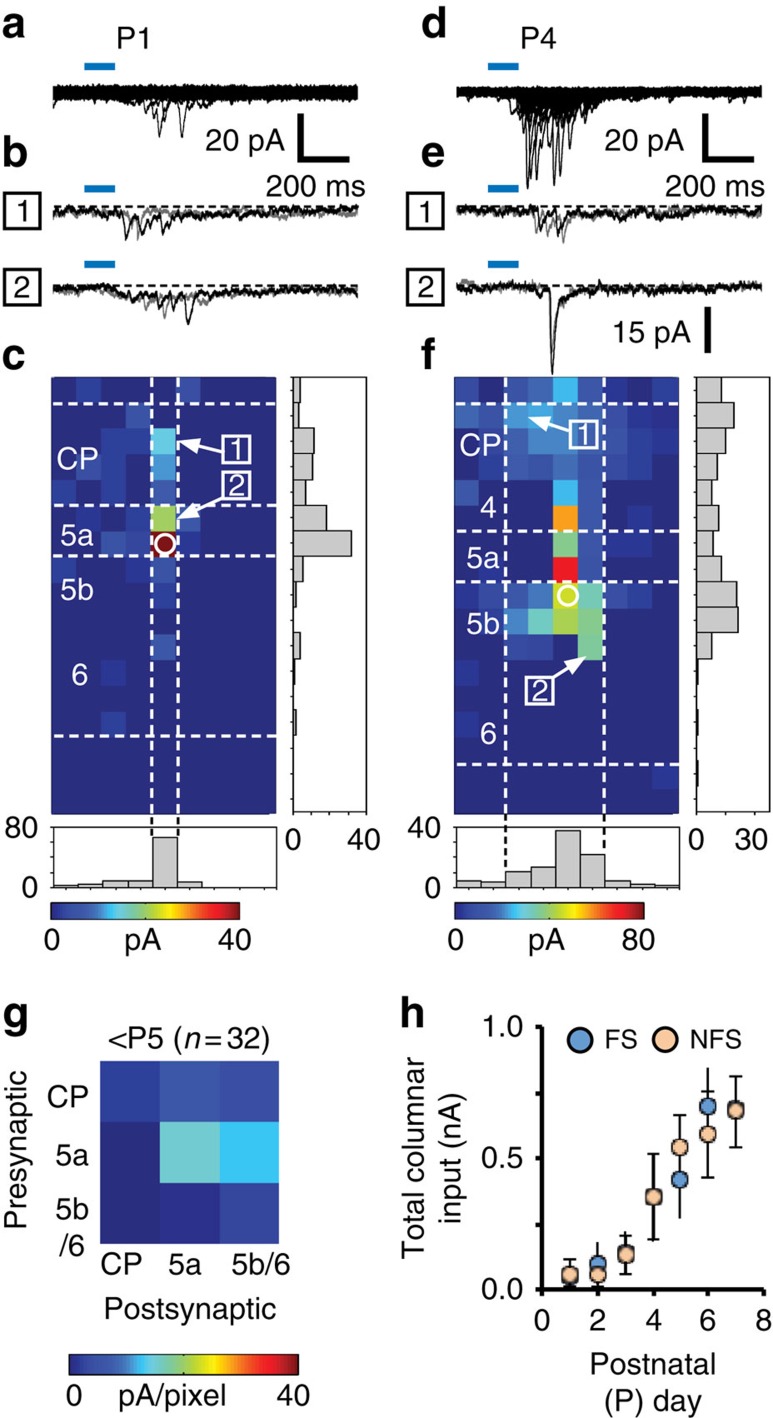
Early onset of glutamatergic afferent input onto *Nkx2-1* interneurons. (**a**) Superimposed voltage clamp sweeps showing the total observed postsynaptic responses recorded onto a P1 immature FS interneuron during LSPS mapping of glutamatergic afferent input across a 153 (9 × 17) spot grid spanning the depth of the neocortex; laser pulse duration indicated by the blue line. (**b**) Superimposed postsynaptic responses observed after repeat photostimulation at the two laser target spots indicated on the average map (**c**). (**c**) Map of synaptic responses (average of *n*=4 maps) for the immature L5a FS cells (position indicated by the open white circle) shown in **a**,**b**. Grey histogram plots show the normalized glutamatergic afferent input across the horizontal (columnar) and vertical (laminar) axes. Horizontal dashed white lines indicate layer boundaries to the nearest 50 μm. Vertical dashed white lines indicate the region of interest (ROI) for subsequent data analysis. (**d**–**f**) Corresponding data for a P4 border L5a/5b NFS interneurons: (**d**) total observed postsynaptic responses recorded across the 153 spot LSPS grid, (**e**) example postsynaptic responses including occasional large (>15 pA) EPSCs and (**f**) map of afferent input onto the NFS interneuron (average of *n*=5 maps). (**g**) Connectivity matrix showing the glutamatergic afferent input onto P1–4 Nkx2-1 interneurons. (**h**) The total columnar (ROI) glutamatergic input onto immature FS (blue circles) and NFS (orange) interneurons over the first postnatal week; all values mean±s.e.m.

We used LSPS with caged glutamate (Supplementary Fig. 2) to map excitatory synaptic afferents onto *Nkx2-1* interneurons throughout development. In contrast to the late emergence of excitatory synaptic connections between immature PYRs^30^, we could elicit reliable, LSPS-evoked excitatory postsynaptic currents (EPSCs) onto *Nkx2-1* interneurons from the first day of recording (P1) onwards (Fig. 4). The presence of the GABA_A_ receptor antagonist picrotoxin (50 μM) had no effect on the optically elicited EPSCs, confirming their glutamatergic nature (*n*=4).

Early (<P5) inputs shared a number of features irrespective of interneuron subtype: first, the majority of *Nkx2-1* interneurons that acquired input before P5 (*n*=32) were located on the layer 5a/5b border (Fig. 4a–f)^16^. Second, PYRs in the cortical plate formed translaminar synaptic connections onto deeper layer interneurons before supragranular layer formation was complete (for example, Fig. 4e,f), although the strongest input onto these cells came from the immediate vicinity (Fig. 4g). Third, *Nkx2-1* interneurons in the cortical plate rarely exhibited LSPS-evoked EPSCs (2/16 cells; Fig. 4g). Fourth, EPSCs were relatively sparse before P4 but prominent thereafter (Fig. 4h), coincident with the emergence of the somatosensory whisker barrel field (S1BF) cytoarchitecture. From P5 onwards, we could directly compare LSPS-evoked glutamatergic input onto *Nkx2-1* interneurons with that onto PYRs^30^ (Supplementary Fig. 3a–c). EPSC amplitudes onto *Nkx2-1* interneurons (NFS mean: 10.7±0.3 pA; sampled from *n*=5 cells; FS: 9.4±0.2 pA; *n*=5; Supplementary Fig. 3d) were larger than EPSCs onto PYRs (mean: 6.9±0.2 pA; *n*=8 cells; Kolmogorov–Smirnov test, *P*<0.05). In addition, LSPS-evoked EPSCs onto *Nkx2-1* interneurons were extremely reliable (Supplementary Fig. 3a,e), a property not seen in PYR–PYR connections^30^. These findings suggest that *Nkx2-1* interneurons—particularly, those in layer 5—integrate early into PYR networks and acquire functional synapses as the cytoarchitecture of the barrel cortex emerges.

### Variable input onto *Nkx2-1* cells through development

**Figure 5:**
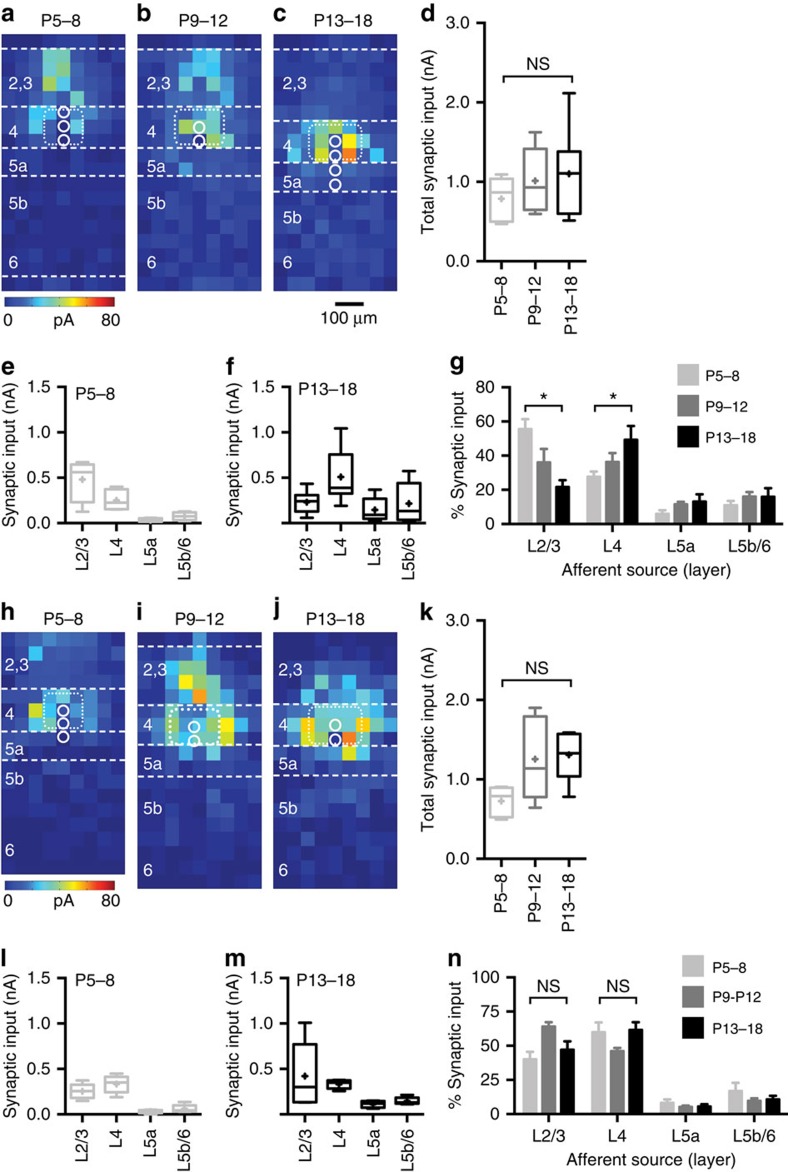
Development of glutamatergic afferent input onto L4 *Nkx2-1* interneuron subtypes. (**a**–**c**) The source of glutamatergic afferent input onto layer 4 (L4) motif FS interneurons collapses into the immediate barrel (average barrel location indicated by the dotted white line) over development (P5–8: *n*=5 cells; P9–12: *n*=6; P13+: *n*=7). The location of the FS interneurons averaged for each map are represented with the open white circles—please note that a single circle on the average map can represent multiple cells recorded at the same corresponding location; horizontal dashed white lines indicate layer boundaries to the nearest 50 μm. (**d**) Total glutamatergic afferent input onto L4 FS cells through early development. Boxplot: cross, mean; horizontal line, median; box, s.d.; error bars, spread of the data; NS, no significant difference between age groups. (**e**,**f**) Breakdown of total input onto L4 FS cells by layer at early (P5–8) and late (P13–18) time points; format of the boxplot as in **d**. (**g**) Percentage afferent input from each layer onto L4 FS interneurons over development; error bars, s.e.m., asterisk, L2/3: *P*=0.004, Kruskal–Wallis test (Dunn correction for multiple comparisons); L4: *P*=0.030 Kruskal–Wallis test (Dunn correction). (**h**–**n**) Corresponding data for L4 NFS interneurons (P5–8: *n*=4 cells; P9–12: *n*=5; P13+: *n*=5).

**Figure 6:**
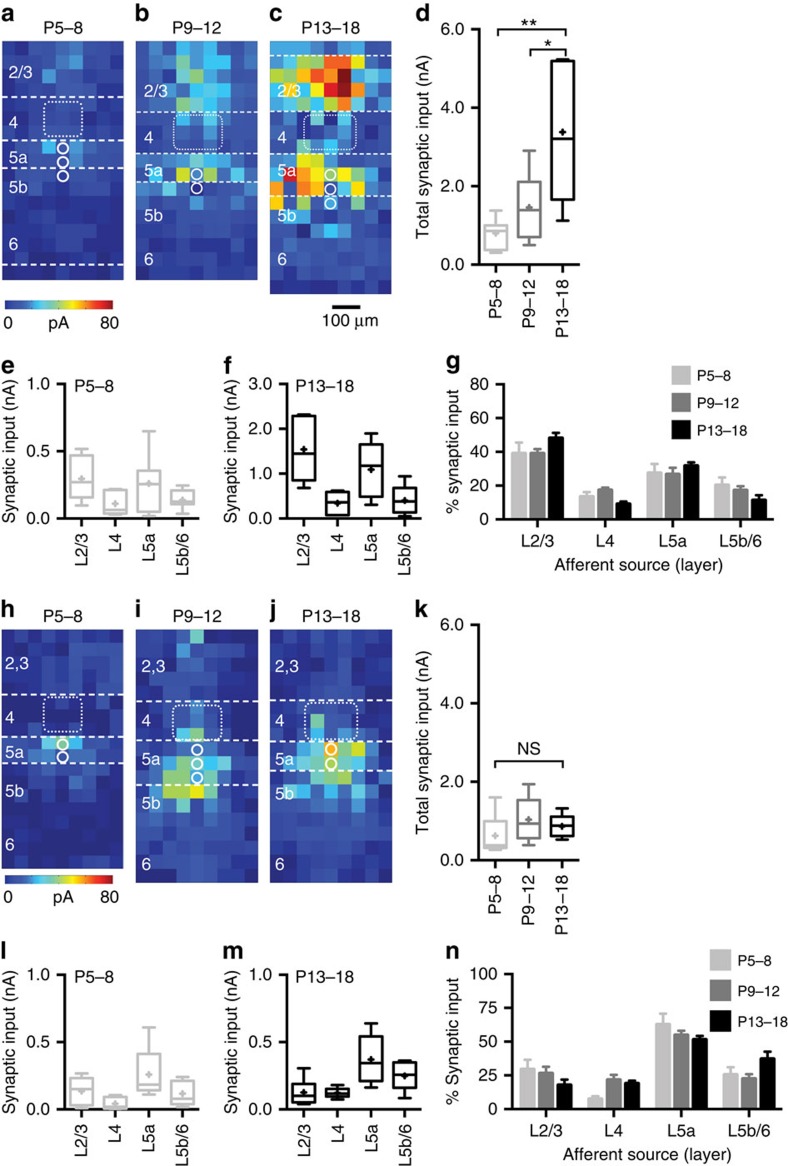
Development of glutamatergic afferent input onto L5a *Nkx2-1* interneuron subtypes. (**a**–**c**) Static motif observed in L5a FS interneurons that underwent little change in the distribution of the source of their afferent glutamatergic input over development. (**a**) P5–8: *n*=7 cells; (**b**) P9–12: *n*=6; (**c**) P13+: *n*=5. (**d**) Total glutamatergic afferent input onto L5a FS cells through early development. Boxplot: cross, mean; horizontal line, median; box, s.d.; error bars, spread of the data; **P=0.005, Mann–Whitney *U*=1; *P=0.045, Mann–Whitney *U*=6). (**e**,**f**) Breakdown of total input onto L5a FS cells by layer at early (P5–8) and late (P13–18) time points; format of the boxplot as in **d**. (**g**) Percentage afferent input from each layer onto L5a FS interneurons over development. (**h**–**n**) Corresponding data for L5a NFS interneurons (P5–8: *n*=6 cells; P9–12: *n*=4; P13+: *n*=4); NS, no significant difference between age groups.

To track the subsequent maturation of glutamatergic inputs onto *Nkx2-1* interneurons, we obtained LSPS maps of 73 FS and 56 NFS *Nkx2-1* interneurons across all cortical layers from P5 to 21. The analysis period was divided into three windows: P5–8, the peak critical period in layer 4 S1BF^31^; P9–12, the period of maturation of the glutamatergic network; and P13+, the period of synaptic consolidation in the juvenile network. For each individual cell, we calculated the absolute (pA/pixel; Supplementary Fig. 2j) and normalized (%pA/pixel; Supplementary Fig. 2k) afferent input from each layer. This allowed us to assign cells to a particular connection motif according to interneuron subtype, afferent input profile, and—to a lesser degree—cell body location. Comparisons of average motifs within (Supplementary Fig. 2j–l) and across epochs (Figs 5 and 6; Supplementary Figs 4 and 5) could identify temporal changes in glutamatergic input.

Consistent with our current understanding of mature cortical networks^20^,^32^, the dominant feature was local innervation of *Nkx2-1* interneurons by glutamatergic PYR neurons. However, some interneurons also received translaminar excitation whose source layer varied during development. FS interneurons located in L4 received glutamatergic afferent input from across L2–4 before the end of the L4 critical period plasticity (P5–8; Fig. 5a). Thereafter, afferent input from supragranular layers weakened (Fig. 5b) and finally disappeared (Fig. 5c).Whereas the total amount of glutamatergic input onto L4 FS cells remained constant over this period of time (Fig. 5d), the distribution of inputs among cortical layers clearly changed from early (Fig. 5e) to late time points (Fig. 5f): translaminar innervation from L2/3 PYRs decreased, while local innervation by L4 spiny stellate neurons increased (Fig. 5g). We refer to this developmental progression as a dynamic integration strategy.

In contrast to L4 FS cells, SST-positive NFS interneurons (Fig. 5h–n) received diffuse input from across L2–4 throughout the time period studied (Fig. 5h–j)^33^. While these interneurons received levels of total glutamatergic input similar to that observed in L4 FS cells (Fig. 5k), the sources of afferent input did not progressively concentrate in L4 (Fig. 5l-n). We refer to this as a static integration strategy. *Nkx2-1* interneurons in L2/3 (Supplementary Fig. 4) and 5a (Fig. 6) also followed static integration strategies, irrespective of subtype. However, in these layers total glutamatergic input onto FS interneurons, but not onto NFS interneurons, progressively strengthened (see for examples L5a FS cells, Fig. 6a–d,h–n).

**Figure 7:**
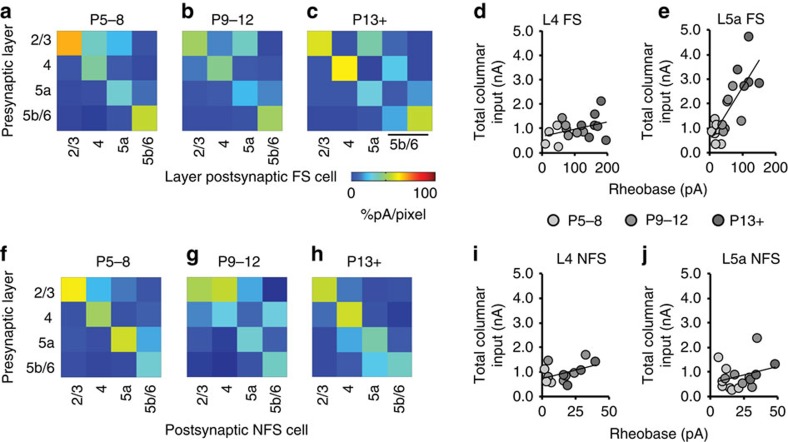
Organization of glutamatergic afferent input onto Nkx2-1 interneurons across the depth of the cortex and the relationship between intrinsic maturation and PYR network integration in developing *Nkx2-1* interneuron subtypes. Connectivity matrices for developing FS interneurons recorded across the depth of S1BF divided according to age: (**a**) P5–8, (**b**) P9–12 and (**c**) P13+. To enable comparison across ages groups, data shown in **a**–**c** are normalized (%pA/pixel). At P13+, an additional L5b/6 motif with prominent afferent input from layer 4 (L4) was observed. (**d**,**e**) Plot of rheobase versus total columnar afferent input for interneurons recorded over development. (**d**) L4 FS interneurons showed little relationship between intrinsic excitability (rheobase) and total columnar input (*R*^2^=0.14), whereas this was more prominent in L5a FS cells (*R*^2^=0.62). (**f**–**h**) Connectivity matrices (%pA/pixel) for immature NFS interneurons recorded across the depth of S1BF; (**f**) P5–8, (**g**) P9–12 and (**h**) P13+. Little relationship was observed between intrinsic properties and network integration either (**i**) L4 (*R*^2^=0.20) or (**j**) L5a (*R*^2^=0.08) NFS interneurons.

Connectivity matrices (Fig. 7) showed that excitatory connections onto FS interneurons develop in a precise manner across the depth of the cortical column. The notable features of this progressive organization are the strength and distribution of connections formed by early supragranular PYRs onto FS cells spanning L2–5a (Fig. 7a,b); the stability of these connections in layers targeted by paralemniscal thalamic afferents^34^; and the contrasting alteration of these connections in lemniscal target layers (Fig. 7c). Changes that occured post-L4 critical period plasticity include the emergence of afferents from L4 onto a subpopulation of L5b/6 FS interneurons that had hitherto been dominated by local connections (Supplementary Fig. 5d)^35^. The increase in glutamatergic input onto FS cells parallels the maturation of the intrinsic properties of this interneuron subtype. The strongest correlation between intrinsic maturation and columnar glutamatergic network integration was found in L5a FS cells (Fig. 7e). These interneurons were dominated by feed-forward (L2/3) and local (L5a) excitation from within the immediate S1BF column across development (Fig. 6a–c,g). In contrast, the total columnar input onto L4 FS cells did not increase over development (Fig. 7d). Two factors likely contribute to this difference with respect to FS cells in other layers: first, early L2/3 PYR input onto L4 FS cells is progressively pruned (Fig. 5a–c,g); and second, L4 FS cells receive strong afferent input from thalamic afferent fibres from P6/P7 onwards^29^. This input was excluded from our strictly columnar analysis.

Transient translaminar connections onto NFS interneurons were observed originating from L2/3 (Fig. 7f,g), alongside a prominent input from L4 onto L5b during the critical period (Fig. 7g; Supplementary Fig. 5e). All these connections disappeared by P13+ (ref. 21; Fig. 7h). In contrast to FS interneurons, the total glutamatergic input onto NFS cells did not increase during development: irrespective of layer, there was no clear developmental relationship between maturation of intrinsic properties and network integration in NFS cells (Fig. 7i,j).

Together, these data highlight a number of layer and cell-type-specific differences in the nature of early PYR-to-interneuron connectivity. We have identified dynamic and static translaminar inputs onto FS interneurons that accompany the emergence of mature cortical circuitry. Changes in connectivity between PYRs and NFS interneurons are less stereotyped than those between PYRs and FS interneurons and dominated throughout early development by local afferents.

### Optogenetic connectivity mapping during development

**Figure 8:**
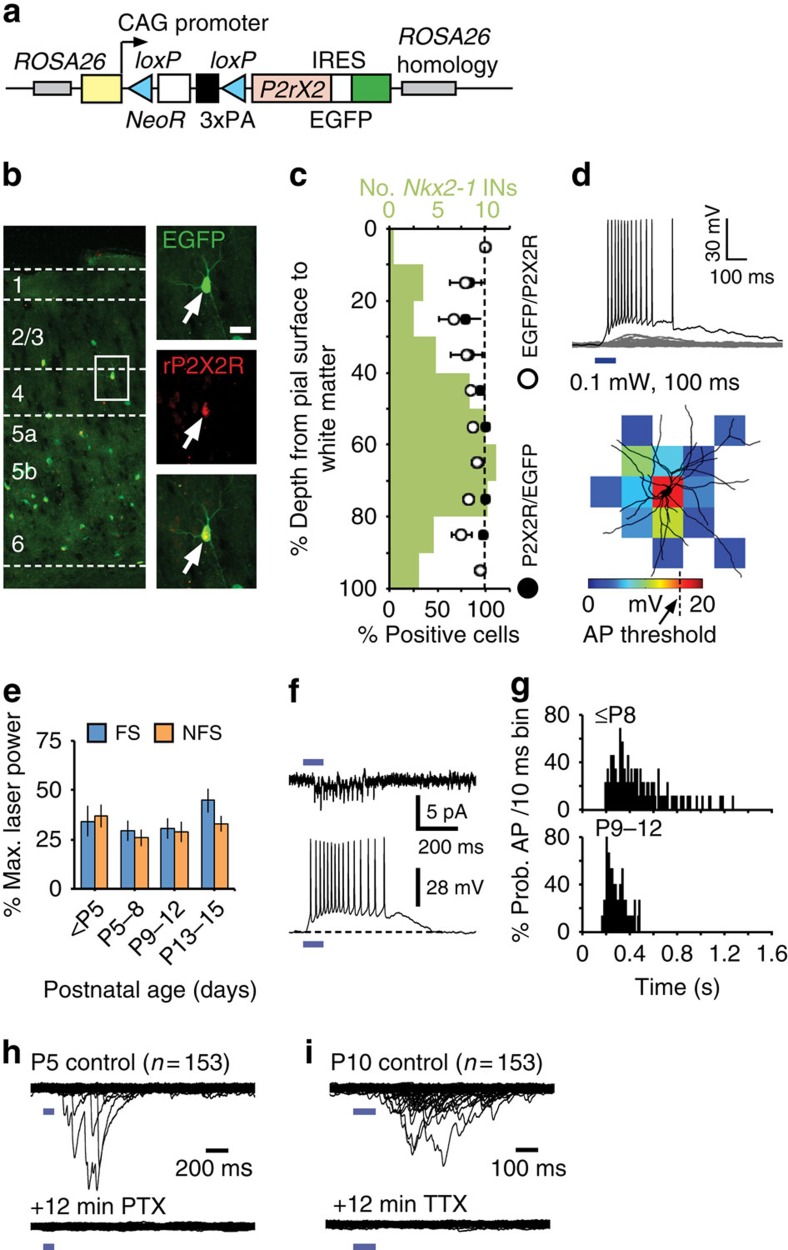
Cell-specific LSPS using conditional expression of the rat ionotropic P2X2 purinergic receptor. (**a**) R26::P2X2 allele construct. Grey boxes, Rosa26 homology sequences; yellow, CAG hybrid promoter; blue triangles, loxP sites; red, open-reading frame; white, selection marker. (**b**) Conditional expression of the rat P2X2 receptor (rP2X2R) in *Nkx2-1* interneurons. Inset top: an EGFP+ cell (arrow) showing expression of rP2X2R (middle); inset bottom: combined image; scale bar, 15 μm. (**c**) Distribution of EGFP+ cells across the depth of the cortex (green histogram bars) with corresponding percentage of EGFP+/P2X2R+ (white circles) and P2X2R+/EGFP+ (black circles). (*n*=631 cells; *n*=5 brains, P7–8). (**d**) Laser intensity was adjusted to give focal ATP uncaging an effective resolution of 50 μm. Top panel: recording across our standard LSPS grid (*n*=153 laser target spots) with only one suprathreshold response (black trace) observed when firing the laser directly at the recorded interneuron soma; blue line, ultraviolet laser. Bottom panel: the dendritic morphology of the same cell superimposed with observed depolarisation colour coded according to the scale bar shown below. (**e**) The average laser power necessary to evoke a consistent, focal presynaptic response over the period of early development studied; Student's *t*-test P13–15, FS versus NFS *P*=0.264, t(10)=1.1828). (**f**) Direct suprathreshold responses recorded in cell-attached (top trace) and whole-cell patch-clamp (bottom trace) configuration (*n*=5 cells). (**g**) The average time course of ATP-evoked presynaptic APs in response to calibrated ultraviolet laser pulses at early (top graph; *n*=7 cells) and late (bottom; *n*=6) ages. (**h**) Top trace: synaptic currents recorded across the extent of the LSPS grid in a P5 PYR neuron in the presence of glutamatergic antagonists CNQX and AP-5 (both 30 μM); bottom trace: GABA_A_ receptor antagonist picrotoxin (50 μM) abolished laser-evoked PSCs. (**i**) Postsynaptic currents in a P10 PYR cell were abolished following incubation with 1 μm TTX (bottom trace).

Mapping of glutamatergic inputs onto *Nkx2-1* interneurons revealed a precise temporal and spatial engagement of FS and, to a lesser degree, NFS populations by early postnatal PYRs. Interneurons form transient networks during development that are distinct from those found in the juvenile and adult brain. To test whether reciprocal connections from *Nkx2-1* interneurons to PYRs followed a similar developmental sequence, we developed an optogenetic approach to selectively map the efferent connections of *Nkx2-1* interneurons. This involved conditional expression of the ionotropic rat P2X2 receptor (P2X2R) from a floxed transgene^24^,^36^ (Fig. 8a) in *Nkx2-1iCre;Z/EG;P2X2* mice. To exclude the possibility of cross-talk via endogenous ATP-gated ion channels, we focally uncaged ATP while recording from either EGFP+ or non-EGFP cells in slices from control *Nkx2-1iCre;Z/EG* animals. Before P14 ATP uncaging elicited neither direct currents nor time-locked postsynaptic currents (PSCs) in these control mice (*n*=28 cells). Immunohistochemistry confirmed the absence of endogenous P2X2R in S1BF at early time points (≤P14), while at P17 P2X2R expression was observed in L5 PYRs in control tissue. In contrast, *Nkx2-1iCre;Z/EG;P2X2* mice exhibited strong rat P2X2R expression, which was confined to EGFP+ cells across the depth of the cortex (Fig. 8b,c).

Photolysis of caged ATP near non-EGFP cells (*n*=52) in early postnatal *Nkx2-1iCre;Z/EG;P2X2* mice failed to elicit direct responses, while aiming a focused laser beam at EGFP+ somata in the same slices resulted in prolonged suprathreshold depolarization, irrespective of *Nkx2-1* interneuron subtype (Fig. 8d; Supplementary Fig. 6a–g). We tested a range of laser settings to establish parameters that afforded consistent levels of activation (Supplementary Fig. 6a–g; Supplementary Table 4) at a spatial resolution of ≤50 μm (Fig. 8d). This resolution could be achieved for all interneuron subtypes without varying laser power throughout early development (≤P12; Fig. 8e). The temporal fidelity of ATP-evoked action potentials, which was similar in cell-attached and whole-cell configurations (Fig. 8f), increased over development (Fig. 8g). To establish the detection window for putative monosynaptic GABAergic inputs^30^, we recorded light-evoked PSCs in PYRs using a high intracellular chloride electrode solution in the presence of glutamatergic antagonists to reduce polysynaptic transmission and block spontaneous EPSCs. The observed temporal profile of PSCs (Supplementary Fig. 6h,i) enabled us to then define the laser-evoked putative monosynaptic event window throughout development and selectively map emergent *Nkx2-1* interneuron input onto recorded PYRs using a strategy similar to that employed for standard caged glutamate LSPS (Supplementary Fig. 6j–m). Finally, we used pharmacology to confirm that the observed postsynaptic events were GABAergic (Fig. 8h; *n*=4 cells) and dependent on light-evoked presynaptic action potentials (Fig. 8i; *n*=3 cells). These data confirm that uncaging of ATP in conjunction with conditional expression of the rat P2X2R provides an effective means for mapping the extent of GABAergic interneuron innervation in the developing neocortex.

### A translaminar shift in the output of a subset of *Nkx2-1* cells

**Figure 9:**
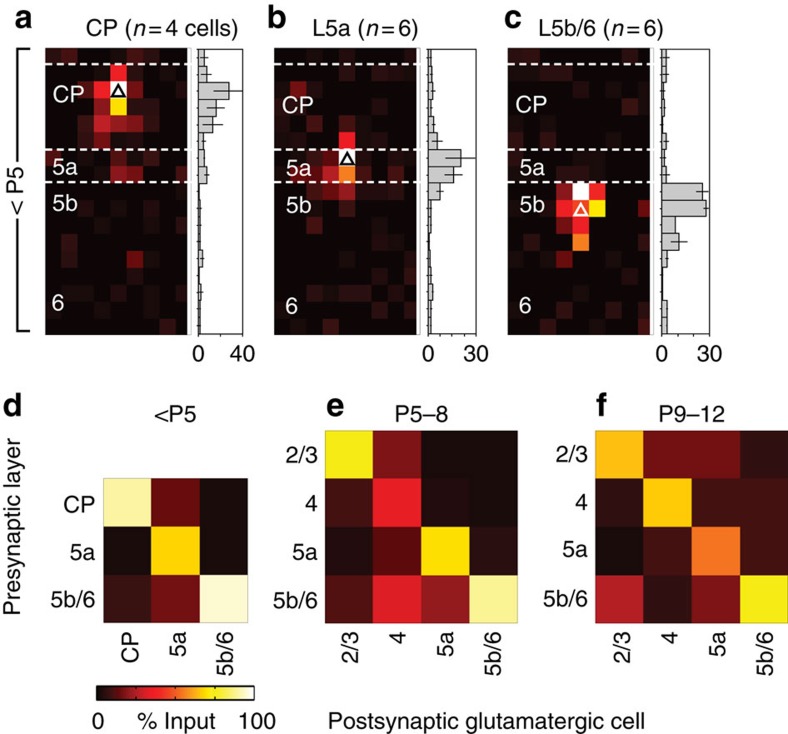
Efferent targets of *Nkx2-1* interneurons are predominantly local in developing S1BF. LSPS mapping of input arising from *Nkx2-1* interneurons onto (**a**) cortical plate (CP), (**b**) L5a and (**c**) L5b/6 pyramidal cells (average position represented by the triangles); scale bar: <P5, 0–16% pA/pixel. (**d**–**f**) Connectivity matrices for *Nkx2-1* interneuron efferent targets across early postnatal development: (**d**) less than postnatal day (P)5; (**e**) during L4 critical period plasticity (P5–9; (**f**) post-critical period plasticity (P9–12).

**Figure 10:**
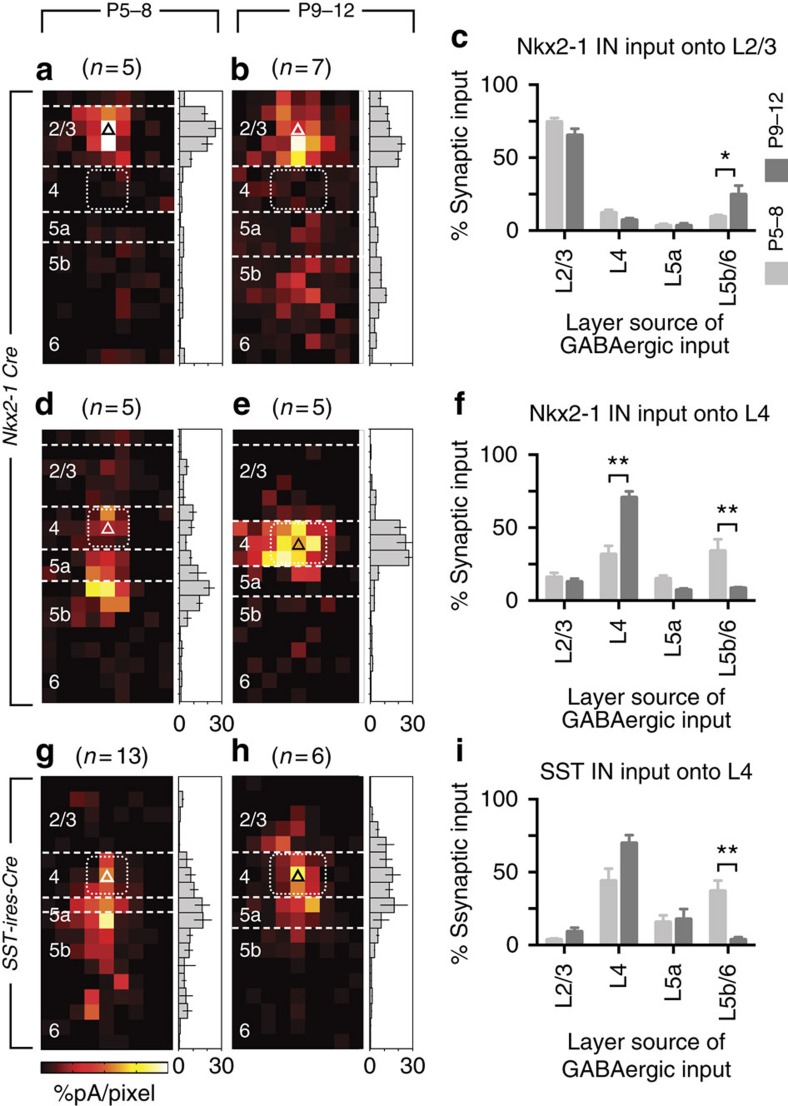
Transient translaminar synaptic connections arising from L5b Nkx2-1 interneuron subtypes. (**a**) During the critical period plasticity L2/3 pyramidal cells received local *Nkx2-1* input but exhibited an increase in L5b input post-critical period (**b**,**c**). *P=0.016, Kruskal–Wallis test, Dunn correction for multiple comparisons. (**d**) L4 neurons received strong L5b input during the critical period that was subsequently (**e**) confined primarily to the immediate barrel. (**f**) Laminar distribution of input arising from *Nxk2-1* interneuron over development; **P<0.001, Kruskal–Wallis test, Dunn correction. (**g**–**i**) LSPS data from L4 glutamatergic neurons in animals in which our optogenetic actuator was bred onto SST-ires-Cre background. (**g**) L5b SST+ cells provide early translaminar input onto L4 cells that is absent post P8 (**h**). (**i**) The laminar distribution of SST+ interneuron input onto L4 PYRs; **P<0.001, Kruskal–Wallis test, Dunn correction. Scale bar, P5–8, 0–10%; P9–12, 0-6%.

PYRs located across the depth of S1BF neocortex received local GABAergic synaptic input from *Nkx2-1* interneurons at the earliest time point recorded (P3; Fig. 9a–c). This pattern of innervation remained intact for infragranular PYRs across the time frame studied (up until P12; Fig. 9d–f; Supplementary Fig. 7). However, L4 and L2/3 PYRs disobeyed this principle in two notable respects (Fig. 10): first, in addition to local inhibition, they received prominent translaminar inhibitory input arising from L5b/6 *Nkx2-1* interneurons. Second, this translaminar inhibition followed a dynamic integration pattern: L5b/6-derived inhibition onto L4 cells was only evident during the critical period of plasticity in that layer (Fig. 10d-f). From P9 onwards, L5b/6-derived inhibition appeared in L2/3 PYRs, which had lacked translaminar input before (Fig. 10a–c). This suggests that L5b/6 interneurons gradually extend their axons from L4 into L2/3 (ref. 32).

The likely mediators of early translaminar innervation of L4 PYRs appeared to be SST+ NFS interneurons within the *Nkx2-1* population, as FS cells develop later^29^ and possess only strictly local axonal arbours at these early ages. To examine this notion, we crossed our conditional P2X2 line with a *SST-ires-Cre* line^37^ to restrict photoactivation to SST+ interneurons. SST+ interneurons in L5b/6 did indeed form transient connections onto L4 cells during the critical period (Fig. 10g), which disappeared thereafter (Fig. 10h,i).

In conclusion, our data reveal the pattern of emergence of *Nkx2-1* interneuron-mediated inhibition across the depth of a cortical column. Using a novel optogenetic strategy, we found that *Nkx2-1* interneurons do not form extensive translaminar connections within the home barrel but rather target local PYRs. The notable exception is L5b/6 *Nkx2-1* interneurons, which provide sequential translaminar innervation first to L4 PYRs and then to L2/3 PYRs. The anatomical substrates of these dynamic ascending connections are NFS, SST+ interneurons.

## Discussion

Exploiting a conditional fate-mapping approach, we identified *Nkx2-1*-derived cortical interneurons from birth and tracked changes in their connectivity across the depth of the cortex throughout early postnatal development. Our data support the idea that GABAergic interneurons are key components of the early neocortex and shed light on the dynamics of interneuron development in a cell-type- and layer-specific manner.

One of the challenges in resolving the contributions of GABAergic interneurons to circuit development is to parse their diversity in the early postnatal cerebral cortex. Genetic strategies^38^, in particular those centred on developmental genetics^15^, have provided a means of tracking cohorts of immature interneurons. One advantage afforded by the *Nkx2-1iCre;Z/EG* line is that it labels remarkably few subtypes of mature interneurons arising from the *Nkx2-1*-expressing ventricular zone of the MGE^7^,^25^,^39^. This specificity is likely due to the absence of *Nkx2-1*-driven *Cre* activity in progenitors within the *Nkx2-1/Nkx6.2* co-expressing region of the VZ in the dorsal aspect of the MGE, a region that has been reported to give rise to a diverse array of interneuron subtypes^40^. Similar to others^29^, we used a combined electrophysiological and histological approach to identify the subtype of fate-mapped EGFP+ interneurons unequivocally.

The next hurdle in understanding inhibitory circuit development is to discriminate the laminar sources of afferent and efferent synaptic connections of developing interneurons. LSPS provides an ideal tool for this purpose: because excitation is confined to the perisomatic (as opposed to axonal or synaptic) compartment, local connections are mapped without interference from fibres of passage^18^,^19^. Our optogenetic strategy has a number of further advantages over previous methods, which make it uniquely suitable to study local circuit connectivity during early development. First, it does not rely on invasive, potentially damaging, shotgun methods such as *in utero* electroporation or neonatal viral transfection. Second, the high single-channel conductance of P2X2 (20 pS)^41^ enables us to evoke suprathreshold currents and drive action potentials in targeted cells at early developmental stages when microbial opsins, which have conductances at least two orders of magnitude smaller^24^, seem ineffective (A.M.-S. and S.B., unpublished observations). Third, in combination with our fate-mapping strategies, the efferent connections of specific cell populations can be resolved through development.

Our knowledge of the role of GABAergic interneurons in early postnatal development has to date focused on two distinct phases: early oscillatory activity^1^ and the critical period for plasticity in L4. Evidence to date assigns these two distinct early developmental roles to different interneuron subtypes—albeit with a common origin in the *Nkx2-1*-expressing MGE. It is evident from our LSPS data that *Nkx2-1* interneurons—irrespective of subtype—initially integrate into the PYR network within the first few postnatal days. In contrast, previous dual recording experiments in visual cortex^16^ succeeded in identifying synaptic connections between interneurons and PYRs only towards the end of the first postnatal week. The early establishment and subsequent growth of synaptic connections between *Nkx2-1* interneurons and PYRs from P4 onwards would allow *Nkx2-1* interneurons to play a key role in early GABA-dependent oscillatory activity in neocortex. Such a role is unlikely to be filled by caudal ganglionic eminence-derived interneurons, which undergo late, activity-dependent maturation^42^,^43^.

At the time that the columnar cytoarchitecture emerges, patterns of synaptic integration of *Nkx2-1* interneurons start to diverge depending on subtype, as well as laminar location. GABAergic activity has been shown to be important for the maturation of glutamatergic synaptic connections^44^,^45^. The formation of specific reciprocal connections between *Nkx2-1* interneurons and PYRs early in development suggests that this connectivity could provide a scaffold for the emergent circuit at a stage when PYR–PYR connections have yet to become firmly established. Indeed, translaminar glutamatergic projections onto FS interneurons are ideally placed to fulfil such a role given that these synaptic connections pre-date the formation of AMPA receptor-mediated contacts between PYRs. This is most evident in the formation of static L2/3 PYR afferent synaptic connections onto L5a FS interneurons at a stage of development when connectivity between PYRs is primarily mediated by silent synapses^30^. This places PV+, FS interneurons at a critical juncture in early PYR–PYR communication. The increase in total input onto FS cells observed in all layers—with the exception of L4—likely reflects the need to control emergent excitation in the developing cortical circuit^46^, much as FS interneurons do in the adult neocortex^47^.

The two morphological cell types in our sample—basket cells with local projections and Martinotti cells with ascending axonal arbours—provide a structural backdrop against which to interpret our optogenetic mapping of early inhibitory connections. As previously reported for adult cortex^32^, local inhibition is dominant during development. This is consistent with the prevalence of local PV+, FS basket cells in cortical interneuron populations in general^48^,^49^ and in our *Nkx2-1* cohort in particular, along with the dense innervation of proximal targets that is characteristic of this interneuron subtype^50^. However, in addition to local inhibition our analysis revealed a number of translaminar inhibitory connections originating in L5b/6. *Nkx2-1* interneurons in this layer innervate L4 cells during the peak critical period of plasticity^31^ and then L2/3 PYRs in the post-L4 critical period^51^,^52^. Among our fate-mapped cohort of interneurons, deep layer SST+ Martinotti cells seemed the most likely anatomical substrate of ascending inhibition, a notion we confirmed by selectively expressing P2X2 in SST+ neurons^37^ and demonstrating that L5b/6 SST+ interneurons are a source of GABAergic input to L4 during the critical period. It remains to be tested if the emergent L5b onto L2/3 input is also mediated by these cells.

Deep layer SST+ interneurons consist of two populations within the *Nkx2-1* cohort: rIB and NFS type-1 subtypes^15^. While IB cells provide local disynaptic inhibition^53^,^54^ via the apical dendritic tuft of deep layer PYRs^55^, NFS type-1 cells share some characteristics with FS basket cells^15^, and as such are analogous to recently described FS mediators of feedback inhibition in the visual cortex^56^,^57^. The timing and sequential targeting of first L4 and then L2/3 PYRs at critical stages of postnatal development suggest that translaminar GABAergic connections could have a role in circuit plasticity^12^,^13^,^47^,^48^. Beyond direct targeting of PYRs, it is possible these cells could facilitate plasticity via disinhibition of PV+ interneurons^49^,^50^, consistent with the reported targeting of the latter cell type by SST+ L5b interneurons (D.L. and S.B., unpublished observations). Intriguingly, the L5b inhibitory pathway fades following early postnatal development, as feedback inhibition from L5b to L2/3 is not a prominent feature of adult somatosensory cortex^32^ yet can be regulated by perturbations in sensory input^58^.

In summary, we find that *Nkx2-1* interneurons, irrespective of subtype, participate in early cortical translaminar synaptic connections with PYRs. However, there is a clear dichotomy between the two principal *Nkx2-1* interneuron subtypes: FS cells function as the primary recipients of translaminar glutamatergic connections, while NFS cells, specifically those in L5b/6, are the main sources of translaminar GABAergic signalling within the cortical column. Our data further show that afferent and efferent synaptic connections of *Nkx2-1* interneurons, once acquired, are neither simply static nor progressively strengthened. Rather, there appear to be tightly choreographed patterns of layer-specific transient innervation, which we have termed dynamic synaptic integration and which may have significant implications for our understanding of both the normal and dysfunctional cerebral cortex.

## Methods

### Generation of a Cre-responsive R26::P2X2R-EGFP allele

The expression of a covalently linked trimer of rat P2X2 was driven by the synthetic *CAG* promoter after Cre-mediated excision of a *loxP*-STOP cassette interposed between transcription and translation start sites. The expression unit was targeted to the *GT(ROSA)26Sor* (*R26*) locus after insertion into the *ROSA26-PA* vector. R1 embryonic stem cells (129 Sv × 129SvJ F1 hybrid) were electroporated with the linearized targeting vectors, and after G418 selection and expansion, homologous recombinant ES cell clones were identified by PCR and confirmed by Southern blotting. Recombinant ES cells were injected into C57Bl/6J blastocysts to produce germline chimeras.

### Animal husbandry and use

The following mouse lines were maintained on an outbred CD1 background: *Nkx2-1iCre* [Tg(Nkx2-1-cre)Kess]; SST-ires-Cre [SSTtm2.1(cre)Zjh]. *Nkx2-1iCre;Z/EG* neonates of both sex (aged between postnatal day (P)1 and 21) were used in experiments. These were generated by breeding *Nkx2-1iCre* hemi-/homozygotes with *Z/EG* (Tg)<CAG-Bgeo/GFP> or *Z/EG*;*R26::P2X2R-EGFP* adults of the opposite sex. Animal care and experimental procedures were approved by the University of Oxford local ethical review committee and conducted in accordance with UK Home Office personal and project (70/6767; 30/3052) licenses under the Animals (Scientific Procedures) 1986 Act.

### Electrophysiology

Whole-cell patch-clamp electrophysiology was performed on acute *in vitro* coronal slices containing S1BF^30^. In brief, mice of either sex were deeply anaesthetized with 4% isoflurane in 100% O_2_ before decapitation and dissection of the brain in ice-cold, artificial cerebral spinal fluid (ACSF; composition (in mM): 125 NaCl, 2.5 KCl, 25 NaHCO_3_, 1.25 NaH_2_PO_4_, 1 MgCl_2_, 2 CaCl_2_, 20 glucose; pH equilibrated with 95%O_2_/5% CO_2_; all chemicals were sourced from Sigma unless otherwise specified). Coronal somatosensory slices (350–400 μm) were cut in ice-cold ACSF using a vibratome before being allowed to recover in ACSF maintained at room temperature for ∼60 min before the onset of recording. Neurons were selected from depths typically >50 μm below the slice surface. Whole-cell patch-clamp recordings were obtained at room temperature using borosilicate glass microelectrodes (Harvard Apparatus, UK) of 6–9 MΩ resistance, pulled using a PC-10 microelectrode puller (Narishige, Japan). Microelectrodes were filled with either a potassium-based (in mM: 128 K-gluconate, 4 NaCl, 0.3 Li-GTP, 5 Mg-ATP, 0.0001 CaCl_2_, 10 HEPES and 1 glucose) or cesium-based internal solution (100 gluconic acid, 0.2 EGTA, 5 MgCl, 40 HEPES, 2 Mg-ATP, 0.3 Li-GTP, 7.2–7.4 pH using CsOH) used to obtain intrinsic electrophysiological properties and LSPS mapping afferent excitatory input, or mapping GABAergic afferent input, respectively. Lucifer yellow or biocytin (∼0.3%) was included to enable morphological reconstruction of the recorded cells. EPSCs were recorded in voltage clamp at −70 mV holding potential. Inhibitory postsynaptic currents (IPSCs) were recorded by voltage clamping the neurons at the reversal potential for glutamate (*E*_glut_). *E*_glut_ was determined empirically by uncaging glutamate proximal to the recorded cell soma and adjusting the holding potential until no net current was observed. Cortical layers were readily distinguished in the infrared-differential interference contrast (IR-DIC) image due to changes in cell size and density. The L4/5a boundary was identified via an abrupt transition from small spherical, densely packed cells to large, pyramidal-shaped sparsely distributed cells in L5a. The L5a/b boundary was apparent through an increase in cell density. As such, under low-magnification IR-DIC imaging, *in vitro* L4 septa and L5b could be observed as a distinct dark bands compared with L5a, which was considerably lighter in tone. Mature interneuron subtypes identified according to established intrinsic electrophysiological, morphological and immunohistochemical criteria^15^.

### Cluster and principal component analysis

The following intrinsic properties were used for cluster analysis: spike threshold (mV), height (mV) and half width (ms); maximum firing frequency (Hz) and spike frequency adaptation; after-hyperpolarisation amplitude (mV) and time to maximum (ms); and input resistance (*R*_IN_, MΩ) and membrane time constant (*τ*). *k*-mean cluster analysis was performed for intrinsic electrophysiological properties with the value of *k* set between 2 and 6. Hierarchical cluster analysis was performed using the Euclidean distance (*eucD*) function and plotted as a dendrogram in Matlab (R2014a). For principal component analysis (PCA), each cell was defined by a string of 4 values that corresponded to the normalized afferent input from L2/3, 4, 5a and 5/6. Analysis was performed in Matlab using the PCA function in the statistics and machine learning toolbox.

### LSPS mapping of glutamatergic afferent input onto interneurons

A glutamate-uncaging LSPS protocol, similar to that previously reported^30^, was used to map afferent synaptic connections onto our *Nkx2-1* interneuron population throughout early postnatal development (Supplementary Fig. 4). Critical to this strategy was use of a long duration (100 ms), low-power (≤2 mW at slice interface) 355-nm ultraviolet laser pulse calibrated to the excitability of PYRs^30^ that enables us to readily parse apart direct (Supplementary Fig. 4a,b) and evoked synaptic responses (Supplementary Fig. 4b,c). This enables the generation of afferent input maps with spatial resolution ∼50 μm throughout the dynamic period of early development. Temporal resolution is maintained by defining a monosynaptic event window^30^ and by repeat mapping of the afferent inputs (Supplementary Fig. 4e). To facilitate the correct assignment of the layers to the result map, a photomicrograph was taken of the low-power IR-DIC image with the overlying target points (Axiovision v4.6 module, UGA-40/UGA-42; Rapp OptoElectronic GmbH, Hamburg, Germany).

### P2X2 optogenetic strategy

Cell-type-selective optogenetic stimulation was performed via expression of the P2X2R in *Nkx2-1* interneurons in *Nkx2-1Cre;Z/EG;R26::P2X2R-EGFP* neonates. Our standard ultraviolet laser LSPS set-up^30^ was used to focally release DMNPE-caged ATP (100 μm; adenosine 5′-triphosphate, P^3^-(1-(4,5-dimethoxy-2-nitrophenyl)ethyl) ester, disodium salt; Life Technologies, UK) across the extent of the target grid. The emitted power of the DPSL-355/30 laser (Rapp Optoelectronic GmbH, Germany) was adjusted to evoke action potentials in *Nkx2-1* interneuron only when focused at the laser target spot immediately above the recorded cell. To establish these setting, the laser was fired repeatedly across the laser target grid, and the laser power adjusted, while recording the interneuron in either loose cell-attached or whole-cell current-clamp configuration. Photostimulation was performed in high divalent ACSF, which included CNQX (30 μm; 6-cyano-7-nitroquinoxaline-2,3-dione; Sigma, UK) and AP-5 (30 μm; DL-2-Amino-5-phosphonopentanoic acid solid; Sigma UK) to block glutamatergic activity. To observe IPSCs in the postsynaptic recorded PYRs, we used a high-chloride intracellular electrode solution (*E*_GABA_∼0 mV)^30^ and voltage-clamped recorded cells at a holding potential of −70 mV.

### Offline analysis of LSPS data

The amplitude of recorded LSPS-evoked and spontaneous EPSCs was extracted offline and compiled to generate maps^30^ (Supplementary Fig. 4e). The resultant grids are then stitched together to capture the entire columnar input (Supplementary Fig. 4f). The region of interest for our subsequent analysis was defined using a threshold of >10% of total input across the columnar (*x*) axis (Supplementary Fig. 4f). While this was an arbitrary threshold, it corresponded closely to the width of the home barrel (following emergence of the barrels at ∼P5; for example, see Supplementary Fig. 4f–i) and enabled us to readily compare cells with different profiles. For each region of interest, we calculated the actual (pA/pixel; Supplementary Fig. 4j) and relative (normalized pA/pixel; Supplementary Fig. 4k) influence of any given layer onto our recorded interneuron. Determining a string of layer-specific values for each mapped cell then enabled us to compare afferent input patterns with different input strengths (pA) across and within a given developmental time frame using PCA (Matlab R2014a; Supplementary Fig. 4l). Motifs were then assigned on the basis of cell type, afferent input and to a lesser degree cell body location, compared through development. Average maps were obtained by aligning the maps according to the L4/5a boundary in the vertical (laminar) axis and by the centre of the home barrel in the horizontal (columnar) axis; layer boundaries were reported to the nearest 50 μm pixel.

### Identification of recorded interneuron morphologies

After completion of the electrophysiology, slices containing Lucifer yellow-filled neurons were transferred to 4% paraformaldehyde in PBS (0.1 M; pH, 7.2) and fixed for a maximum of 3 h, at 4 °C under weighted fine gauze, to prevent distortion of the tissue. The slices were either tested for expression of SST (1:300 rabbit anti-SST; AB5494, Millipore, UK) or the lucifer yellow was converted (1:500 rabbit anti-lucifer yellow biotin-XX conjugate; A-5751 Molecular Probes Inc., UK) to a dense, dark-coloured diaminobenzidine content to facilitate detailed histological reconstruction^26^.

### Tissue preparation and immunohistochemistry

Following terminal general anaesthesia, mice were transcardially perfused with 4% paraformaldehyde in PBS and post-fixed for 1–2 h, depending on neonatal age. Brains were then washed in PBS, cryoprotected by sequential exposure to 10% then 30% sucrose in PBS before being embedded in OCT compound (VWR International Ltd, UK) on dry ice. Tissues were sectioned serially at 14–16 μm and mounted on SuperFrost Plus slides (VWR International Ltd, UK). Before immunohistochemistry, slides were air dried for a minimum of 3 h before being washed with PBS. Tissue was blocked with 10% heat-inactivated normal goat serum (NGS) and 0.5% Triton-X (TX; Sigma, UK) for 2 h, before being incubated in primary antibody (in PBS and NGS/TX) overnight at 4 °C. The following primary antibodies were used: rabbit anti-SST (1:600, AB5494, Millipore), rat anti-SST (1:400, MAB354, Millipore), mouse anti-PV (1:400, MAB1572, Millipore); rabbit anti-P2X2 receptor (extracellular; 1:400 detects rat and mouse; APR-025, Alomone labs, Israel), rabbit anti-P2X2 receptor (1:400, detects mouse; ab48864, Abcam plc, UK), rabbit anti-Nkx6.2 (1:600, ab58708, Abcam plc), chicken anti-GFP (1:400, ab13970, Abcam plc), rabbit anti-calretinin (1:600, AB5054, Millipore) and mouse anti-calretinin (1:400, MAB1568, Millipore). Before incubation with the relevant secondary antibody (1:200; fluorophores: Cy2 (ab6960, ab6952), Cy3 (AP183C, AP132C), Cy5 (ab6563, ab6565), AMCA (AP181M); Abcam plc/Millipore), slices were washed thoroughly in PBS. Subsequent to completion, slides were washed once more, mounted in fluorescent mounting medium, permanently sealed using coverslips with nail polish and imaged (Leica TCS SP5 confocal microscope/Leica DMR microscope). Exposure times were individually set for each image depending on signal strength and the wavelength of the secondary antibody. Further offline editing (Adobe Photoshop CS6) was only done to brightness and contrast levels, and was applied for the entire frame.

### Data sampling and statistical analyses

*Electrophysiology*. cells were recorded across the depth of the S1BF cortex for slices obtained from any given brain to avoid repeat sampling from within the same layer. EGFP+ cell bodies were targeted in a random manner. All cells recorded that were found on switching to lower power IR-DIC imaging to be in vertical alignment with the barrel septa have been excluded from the subsequent analysis. *Immunohistochemisty*: to determine the percentage marker expression in the total EGFP+ population, cell counts were performed across the depth of an arbitrary 250 μm column aligned over S1BF. Cells counts were performed on tissue samples obtained from four to nine pups at each age group that were obtained from three *Nkx2-1iCre;Z/EG* litters from independent adult breeding pairs. All statistical analysis was performed in Matlab R2014a. Data are present as mean±s.e.m. Statistical significance was tested using a Student's *t*-test, the α-level was set at 0.05.

## Additional information

**How to cite this article:** Anastasiades, P. G. *et al*. GABAergic interneurons form transient layer-specific circuits in early postnatal neocortex. *Nat. Commun.* 7:10584 doi: 10.1038/ncomms10584 (2016).

## Supplementary Material

Supplementary Information Supplementary Figures 1-7 and Supplementary Tables 1-4
