# Supplementary material for: GABAergic interneurons form transient layer-specific circuits in early postnatal neocortex
Source: Nat Commun. 2016 Feb 4;7:10584. doi: 10.1038/ncomms10584 (PMC4743032; doi:10.1038/ncomms10584)
Supplement: Supplementary Information — Supplementary Figures 1-7 and Supplementary Tables 1-4 [file ncomms10584-s1.pdf]

GABAergic interneurons form transient layer-specific circuits in early postnatal neocortex Anastasiades *et al.*

SUPPLEMENTARY INFORMATION

Supplementary Figure 1.

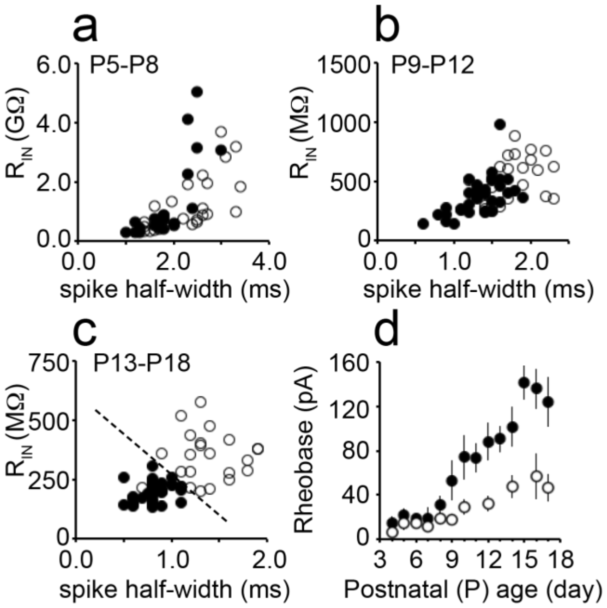

Supplementary Figure 1. Progressive maturation of intrinsic electrophysiological discriminators of *Nkx2-1* interneuron subtypes through development. (a-c) Plots of spike width at half amplitude (spike half-width) versus input resistance (R_{IN}) over development reveal the progressive emergence of the two distinct subtypes ; FS cells, black circles; NFS, white circles. (d) Plot of average rheobase (minimal current to evoke an action potential) for FS (black circles) and NFS (white circles) over development.

Supplementary Figure 2.

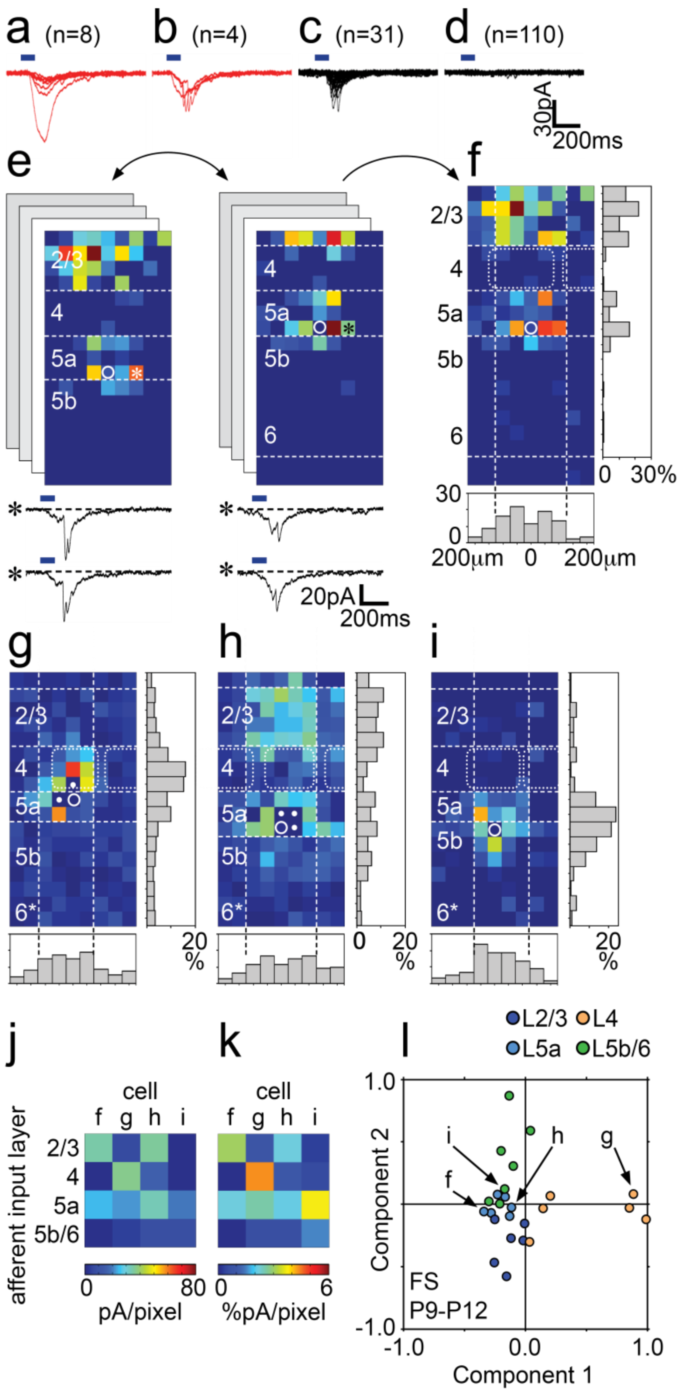

Supplementary Figure 2. LSPS strategy used to map glutamatergic afferent input onto *Nkx2-1* interneurons through early development. A calibrated UV laser pulse (duration shown by thick blue line above the traces) evoked either direct responses (shown superimposed in red)(a) without or (b) with superimposed synaptic responses, (c) synaptic responses alone or (d) no response when fired across a single LSPS grid (n=153 spots). (e) The sum amplitude (pA) of evoked EPSCs plotted for each spot across the grid. The position of the grid was adjusted to ensure that the whole depth (*cont.*)

of the neocortex was mapped multiple times; example responses are shown below the maps from the pixel proximal to the cell body marked with an *asterisk*. Dashed white lines, layer boundaries to the nearest 50 μm. (f) The average LSPS map across the entire depth of the cortex for the recorded P12 FS interneuron; histograms: normalized afferent input calculated for both horizontal and vertical arrays; dotted white line, home and adjacent L4 barrels. (g-i) a range of afferent input patterns observed in layer 5a FS interneurons recorded during the same developmental time window (P9-P12); white dots, pixels for which data was not obtained due to excessive direct responses obscuring potential synaptic events. Connectivity matrices plotted for the cells shown in panels (f-i) according to (j) pA/pixel and (k) normalized (%) pA/pixel. (l) Example of the principal component analysis (PCA) used to further investigate LSPS map data. This plot shows for all FS interneurons recorded between P9-P12, color coded according to the layer location of the recorded cell soma. Cells identified by the letters correspond to those shown in panels (f-i). In the P9-12 window there was a clear split between L4 FS interneurons that received only L4 input (clustered around cell 'g') and those that still received prominent L2/3 afferent input.

Supplementary Figure 3.

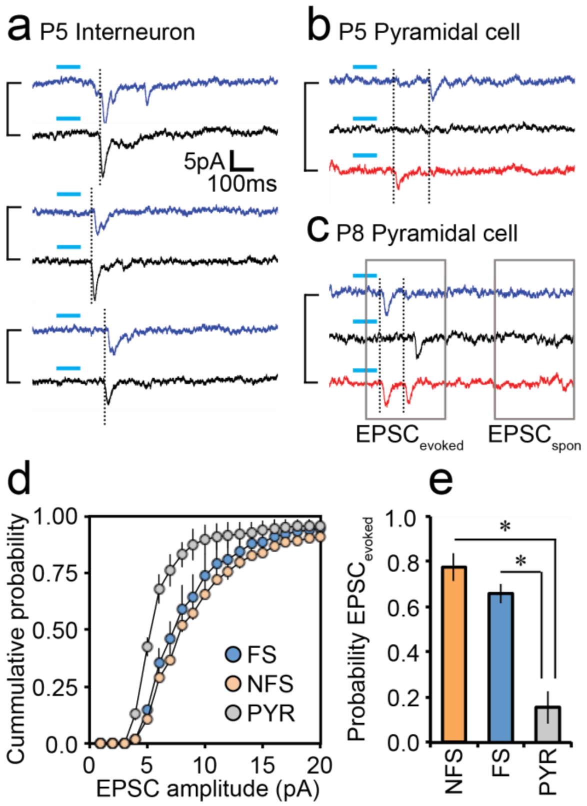

Supplementary Figure 3. Precision of early glutamatergic afferent input onto *Nkx2-1* interneurons within the first few postnatal days. Early glutamatergic afferent input on interneurons (a) differed in both the observed amplitude and efficacy when compared to (b) correspondingly early (P5) PYRs and those recorded (c) at the end of the first postnatal week. The blue line above the individual traces represents the duration of laser firing; brackets indicate paired dashed black line, onset of LSPS-evoked EPSCs. (d) Cumulative probability plot of the EPSC amplitude observed for immature FS (blue circles; n=5 cells recorded in 4 different animals), NFS (orange; n=5 cells, 4 animals) interneurons and PYRs (grey; n=8 cells, 6 animals) mapped at P5. To establish the efficacy of LSPS-evoked synaptic transmission for the various cell types at P5 we calculated the occurrence of spontaneous EPSCs ($EPSC_{spon}$) in an arbitrary time window at the end of our recording trace (see panel (c)). This allowed us to then determine the number of EPSCs within the putative monosynaptic window that were likely to occur purely as a result of LSPS ($EPSC_{evoked}$) (panel (c)) and then establish the probability that an $EPSC_{evoked}$ would be observed after repeat stimulation. (e) Repeat evoked EPSCs were significantly ($P<0.05$ indicated by the asterisks, student's t-test) more likely to occur in NFS (orange; n=5 cells, *vs.* PYR $p<0.001$, $t(8)=8.4561$) and FS (blue; n=5; vs. PYR $P<0.001$, $t(8)=8.4061$) interneurons as opposed to PYR (grey histogram bar; n=5) cells.

Supplementary Figure 4.

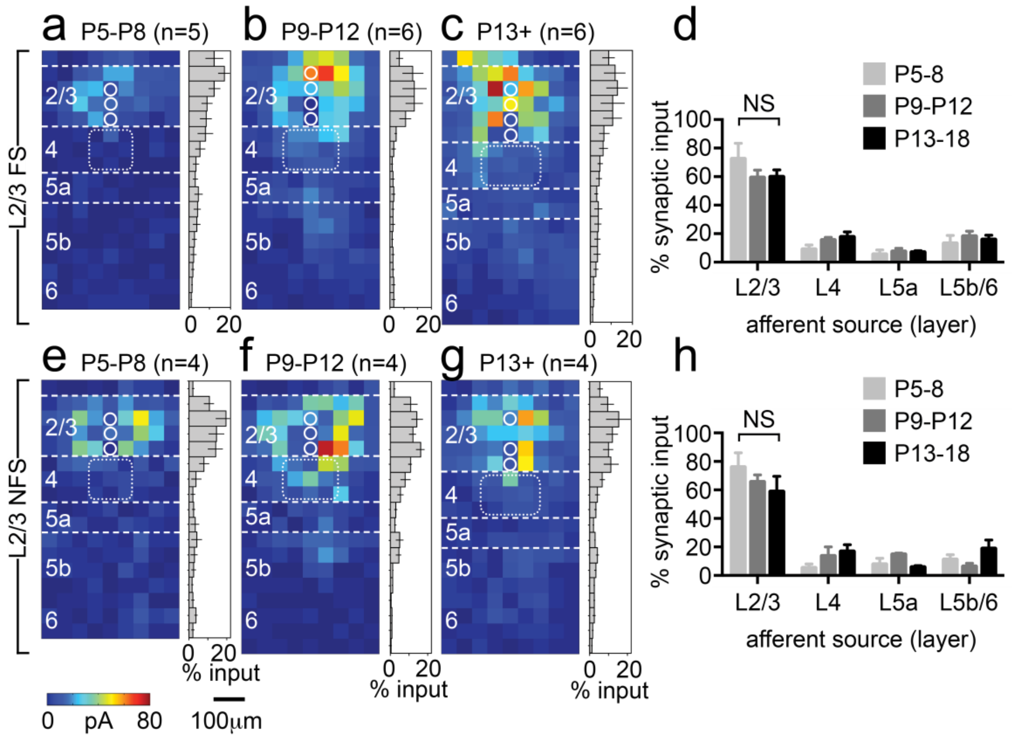

Supplementary Figure 4. Development of glutamatergic afferent input onto layer 2/3 *Nkx2-1* interneurons. (a-c) Development of glutamatergic afferent input onto layer 2/3 FS interneurons. The location of the FS interneurons averaged for each map are represented with the open white circles; horizontal dashed white lines indicate layer boundaries to the nearest 50µm; histograms represent the distribution of the normalised afferent input across the vertical (laminar) axis. (d) Normalised laminar distribution of the afferent input onto L2/3 FS cells through development. (e-h) Corresponding data for layer 2/3 NFS cells.

Supplementary Figure 5.

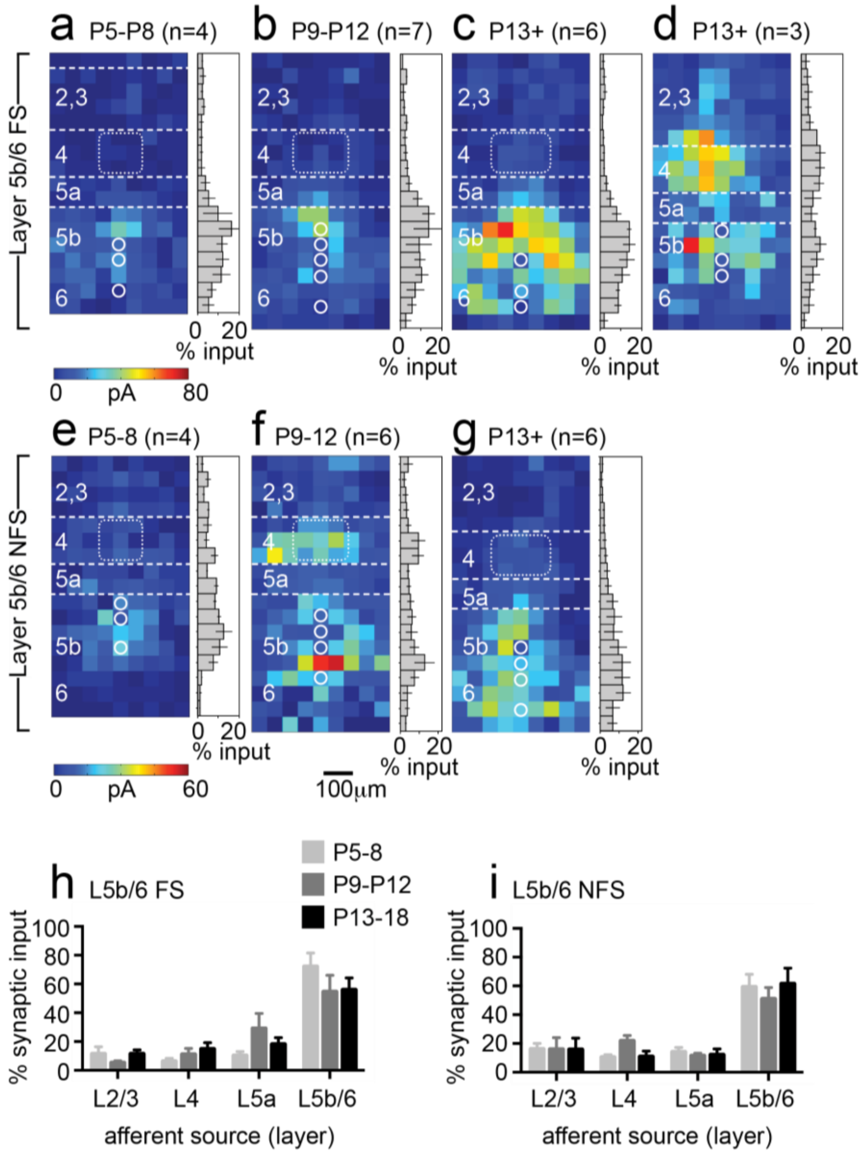

Supplementary Figure 5. Development of glutamatergic afferent input onto layer 5b/6 *Nkx2-1* interneurons. (a-d) Development of glutamatergic afferent input onto layer 5b/6 FS interneurons. The location of the FS interneurons averaged for each map are represented with the open white circles; horizontal dashed white lines indicate layer boundaries to the nearest 50μm; histograms represent the distribution of the normalised afferent input across the vertical (laminar) axis. The P13+ cohort (c,d) was divided due to the absence (c) or presence (d) of input from L4 evident in 3 recorded FS cells at this late time point. (e-g) Corresponding data for layer 5b/6 NFS interneurons. (h,i) Normalised laminar distribution of the afferent input onto L5b/6 (h) FS and (i) NFS cells through development.

Supplementary Figure 6.

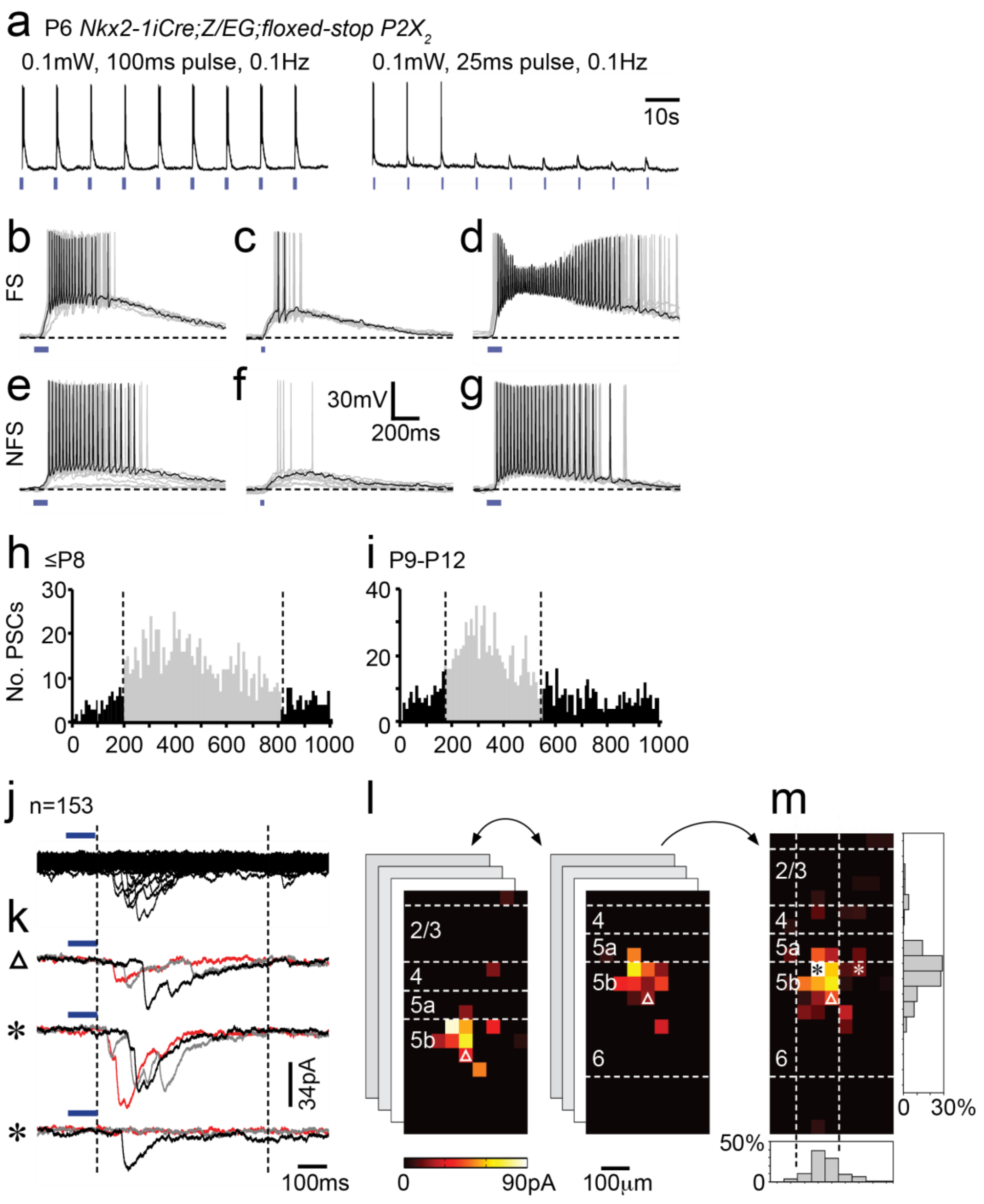

Supplementary Figure 6. High spatial resolution mapping of *Nkx2-1* interneuron input onto postsynaptic pyramidal cells in the early postnatal neocortex. Conditional expression of P2X2 in *Nkx2-1* interneurons allowed us to selectively drive FS and NFS cells using UV laser focal uncaging of ATP in proximity to the recorded cell. The kinetics and efficacy of our novel optogenetic strategy were tested using repetitive stimulation targeted at EGFP+ soma with laser power, pulse duration (a) and frequency of stimulation systematically varied to attain the most consistent levels of stimulation. (b-d) responses in the FS interneuron shown in the left panel (a) observed in response to varied laser parameters. (b) Superimposed traces showing the response recorded following optical (*cont.*)

stimulation using 0.2mW, 100ms, 0.1Hz. (c) The impact of reducing the duration of the pulses from 100 to 25ms. (d) Increasing laser power to 0.4mW greatly reduced the temporal resolution. (e-g) Superimposed traces obtained from repetitively stimulating a layer 5a NFS interneuron: (e) 0.2mW, 100ms, 0.1Hz, (f) 0.2mW, 25ms, 0.1Hz (g) 0.4mW, 100ms, 0.1Hz. (h) Time course of laser-evoked PSCs recorded in PYRs at (h) early (≤P8; n=840 PSCs recorded in 5 cells) and (i) late (P9-12; n=1262 PSCs observed in 4 cells) ages. The start and end of the putative monosynaptic event window are indicated with vertical dashed lines; the number of PSCs observed within that time are shaded grey. (j-m) Cell type specific optogenetic mapping strategy. (j) Superimposed traces showing the observed postsynaptic response to when the laser was fired at all 153 laser target spots; the start and end of the monosynaptic event window are indicated by the vertical dashed lines; the horizontal blue line indicated the duration of the UV laser pulse. (k) Repeat responses observed at single laser target spots. (l) Repeat mapping of the cells was performed, the position of the laser target grid adjusted to ensure that whole depth of the cortex was mapped; the location of the mapped cell is indicated by the white triangle. (m) The average map across the entire depth of the cortex for the recorded layer 5b PYR; histograms: normalized *Nkx2-1* interneuron afferent input calculated for both horizontal and vertical arrays.

Supplementary Figure 7.

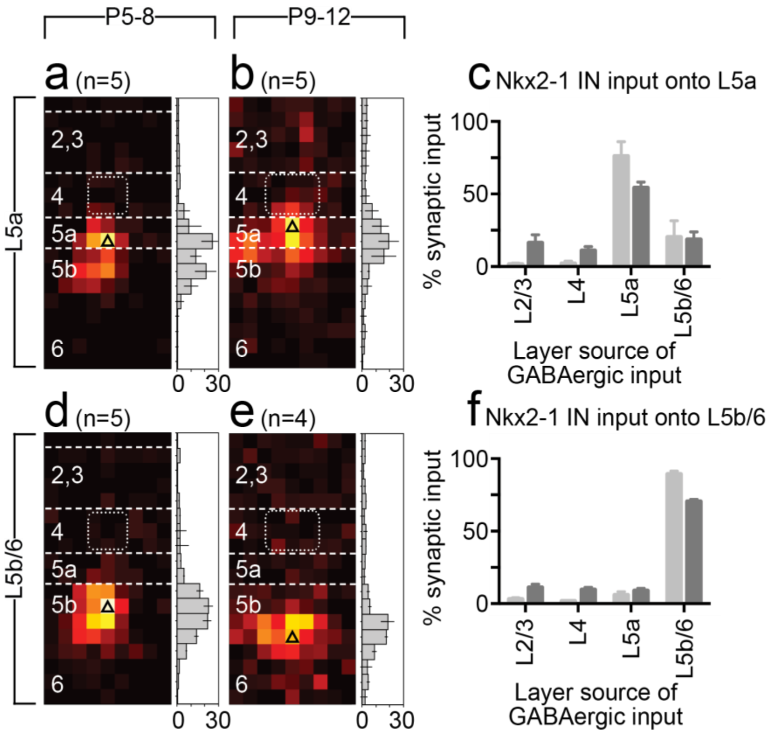

Supplementary Figure 7. Infragranular pyramidal cells receive local inhibition from *Nkx2-1* interneurons throughout early postnatal development.

Nkx2-1 interneuron input onto L5a pyramidal cells from (a) P5 to P8 and (b) P9-P12 is dominated (c) by *Nkx2-1* interneurons located within L5a. (d-f) Local inhibition is also the major source of GABAergic input arising from Nkx2-1 interneurons onto L5b/6 pyramidal cells.

Supplementary Table 1. Intrinsic properties of *Nkx2-1* interneurons P5-P8

Cell type	RMP (mV)	Input resistance (MΩ)	Membrane constant (tau)(ms)	Rheobase (pA)	Spike			Max. firing freq. (Hz)	Adaptation in firing freq. (%)
					Threshold (mV)	Height (mV)	Half width (ms)		
FS (n=31)	-57±7	694±422	48±23	24±17	-35±6	59±6	1.7±0.5	74±16	23±6
NFS (n=25)	-57±5	940±586	57±30	14±9	-43±6	76±12	2.0±0.5	56±10	25±9
rIB (n=2)	-55	708	85	8	-41	75	2.0	50	43

All values ±SD

P<0.05 *vs.* FS   P<0.05 rIB *vs.* NFS

Supplementary Table 2. Intrinsic properties of *Nkx2-1* interneurons P9-P12

Cell type	RMP (mV)	Input resistance (MΩ)	Membrane constant (tau)(ms)	Rheobase (pA)	Spike			Max. firing freq. (Hz)	Adaptation in firing freq. (%)
					Threshold (mV)	Height (mV)	Half width (ms)		
FS (n=41)	-59±6	327±123	30±21	70±47	-34±4	61±8	1.3±0.3	98±30	25±13
NFS (n=27)	-59±5	494±193	40±18	26±16	-42±5	71±8	1.6±0.3	69±22	35±17
rIB (n=6)	-61±2	501±153	38±6	16±7	-48±3	75±11	1.7±0.2	65±10	39±8

Supplementary Table 3. Intrinsic properties of *Nkx2-1* interneurons P13-P18

Cell type	RMP (mV)	Input resistance (MΩ)	Membrane constant (tau)(ms)	Rheobase (pA)	Spike			Max. firing freq. (Hz)	Adaptation in firing freq. (%)
					Threshold (mV)	Height (mV)	Half width (ms)		
FS (n=40)	-62±5	279±187	14±8	117±48	-33±4	58±9	0.8±0.2	157±25	16±10
NFS (n=23)	-58±5	354±103	34±17	51±30	-39±4	71±9	1.4±0.5	70±23	41±11
rIB (n=14)	-59±3	643±132	45±14	13±9	-46±5	72±14	1.4±0.2	72±17	36±8

Supplementary Table 4. Laser parameters for P2X2 optogenetic stimulation of *Nkx2-1* interneurons.

Laser Power (mW)[1]	Pulse duration (ms)	Repetition (Hz)[2]	No. of cells tested (FS:NFS)	No. of evoked APs[3]/laser pulse	% AP failure rate	Observed spatial resolution[4]
0.1	100	0.1	26 (17:9)	15±3	0	≤50µm
0.2	100	0.1	26 (17:9)	16±4	0	≤50µm
0.4	100	0.1	8 (5:3)	45±11	0	>50µm
0.1	25	0.1	10 (6:4)	1±1	74±18	<50µm
0.2	25	0.1	10 (6:4)	2±1	61±23	<50µm
0.4	25	0.1	7 (5:2)	10±4	11±18	≤50µm
0.1	100	0.25	8 (6:2)	7±7	30±19	≤50µm
0.2	100	0.25	8 (6:2)	14±7	0	≤50µm
0.2	25	0.25	3 (3:0)	2±1	57±14	≤50µm

1. Laser power at slice interface measured prior to whole cell patch clamp.
2. Frequency at which the laser was repetitively fired at a single spot targeted at the EGFP+ cell soma.
3. Average number of action potentials (APs).
4. Data obtained from firing the laser across the whole LSPS grid; ≤50µm is equivalent to 1 pixel.

Grey shaded boxes indicate poor temporal and/or spatial resolution
